# Fungal Melanin Biosynthesis Pathway as Source for Fungal Toxins

**DOI:** 10.1128/mbio.00219-22

**Published:** 2022-04-27

**Authors:** Jia Gao, Max Wenderoth, Maria Doppler, Rainer Schuhmacher, Doris Marko, Reinhard Fischer

**Affiliations:** a Karlsruhe Institute of Technology (KIT), Institute for Applied Biosciences, Department of Microbiology, Karlsruhe, Germany; b University of Natural Resources and Life Sciences, Vienna, Department of Agrobiotechnology (IFA-Tulln), Institute of Bioanalytics and Agro-Metabolomics, Tulln, Austria; c University of Natural Resources and Life Sciences, Vienna, Core Facility Bioactive Molecules: Screening and Analysis, Tulln, Austria; d University of Vienna, Department of Food Chemistry and Toxicology, Vienna, Austria; Universidad de Córdoba

**Keywords:** melanin, altertoxin, perylene quinone, Alternaria, mycotoxin, Alternaria alternata, food safety, secondary metabolite

## Abstract

Contamination of food and feed with toxin-producing fungi is a major threat in agriculture and for human health. The filamentous fungus Alternaria alternata is one of the most widespread postharvest contaminants and a weak plant pathogen. It produces a large variety of secondary metabolites with alternariol and its derivatives as characteristic mycotoxin. Other important phyto- and mycotoxins are perylene quinones (PQs), some of which have anticancer properties. Here, we discovered that the PQ altertoxin (ATX) biosynthesis shares most enzymes with the 1,8-dihydroxynaphthalene (1,8-DHN) melanin pathway. However, melanin was formed in aerial hyphae and spores, and ATXs were synthesized in substrate hyphae. This spatial separation is achieved through the promiscuity of a polyketide synthase, presumably producing a pentaketide (T4HN), a hexaketide (AT4HN), and a heptaketide (YWA1) as products. T4HN directly enters the altertoxin and DHN melanin pathway, whereas AT4HN and YWA1 can be converted only in aerial hyphae, which probably leads to a higher T4HN concentration, favoring 1,8-DHN melanin formation. Whereas the production of ATXs was strictly dependent on the CmrA transcription factor, melanin could still be produced in the absence of CmrA to some extent. This suggests that different cues regulate melanin and toxin formation. Since DHN melanin is produced by many fungi, PQs or related compounds may be produced in many more fungi than so far assumed.

## INTRODUCTION

Many secondary metabolites that are used as pharmacological drugs or that are important as food contaminants are of fungal origin (1–3). Secondary metabolites are not only used to treat human and animal infections (e.g., penicillins, griseofulvin) or diseases (e.g., lovastatin) but also as fungicides for plant protection (e.g., strobilurins) (4, 5). Most filamentous fungi produce a blend of different metabolites, for most of which their natural function is still unknown and not so easy to decipher (6, 7). Meanwhile, hundreds of fungal genomes have been sequenced, and most contain a large number of genes encoding enzymes for secondary metabolite biosynthesis (8, 9). However, under laboratory conditions, most secondary metabolite gene clusters are silent. Therefore, many tools and strategies have been developed over the years to assign functions to the *sleeping genes* or to discover novel secondary metabolites (3, 10–13).

Often secondary metabolites are quite complicated chemical molecules, and their biosynthesis requires the coordinated action of several enzymes (1, 14). For many years, it was common knowledge that fungal secondary metabolites were produced by enzymes whose genes are organized in gene clusters where the coordinated expression may be achieved through a transcriptional activator, itself located within the gene cluster (10, 15, 16). The bona fide example is the aflatoxin gene cluster of Aspergillus flavus, which is highly conserved in Aspergillus nidulans (17). However, since the discovery of the biosynthesis of aflatoxin and the corresponding gene cluster, many variations have been described (18). For instance, in the case of meroterpenoids or xanthones, two separate gene clusters are required for their biosynthesis (6, 19). Another example is the nematode-trapping fungus Duddingtonia flagrans, where the expression of some cluster genes is spatially regulated, resulting in different metabolites at different places in the mycelium (20).

The fungus Alternaria alternata is a widespread food contaminant and a weak plant pathogen (21–24). It is also an important allergen (25). A. alternata is known to produce a large number of different secondary metabolites (26–28). For many of them, their biosynthesis route is still unclear, although many are recognized as toxins in food and feed (24, 29). The biosynthesis of the polyketide alternariol and the corresponding gene cluster, *pksI*, were characterized recently (30, 31).

Another important polyketide-type secondary metabolite in fungi is the black pigment dihydroxynaphthalene (DHN) melanin (32). In some fungi, like Aspergillus fumigatus, all genes required for the biosynthesis are clustered, but in A. alternata, only some genes are found in the same genomic region, not all genes have been identified and studied at the molecular level, and the exact pathway for the biosynthesis is still not clear (33–35). For instance, the polyketide synthase (PKS) enzymes responsible for DHN melanin formation from different fungi display sequence similarity, but three different products, namely, 2,5,6,8-tetrahydroxy-2-methyl-2,3-dihydro-4H-naphtho(2,3-b)pyran-4-one (YWA1), 2-acetyl-1,3,6,8-tetrahydroxynaphthalene (AT4HN), and 1,3,6,8-tetrahydroxynaphthalen (T4HN), can be synthesized. A. fumigatus for example, produces the heptaketide YWA1, which is hydrolyzed to the pentaketide T4HN, whereas Botrytis cinerea produces T4HN and the hexaketide AT4HN using two different PKS enzymes (36, 37). AT4HN is then also further converted to T4HN. The specificity of the A. alternata enzyme has not been determined yet.

A. alternata produces also phytotoxins of the perylene quinone (PQ) family, such as altertoxins I, II, III (ATX I, II, and III) and some other derivatives (28, 38, 39). The compounds are also harmful to human, and since A. alternata is a common food contaminant, ATXs represent a severe threat to human health. However, regulations for the control of ATXs in food are still missing. ATX II was reported as a very powerful mutagen and DNA strand-breaking agent in Chinese hamster V79 cells, exhibiting at least 50-fold higher mutagenicity than the best-studied *Alternaria* mycotoxins alternariol (AOH) and alternariol monomethyl ether (AME) (40). ATX II introduces two covalent deoxyguanosine adducts when incubated with DNA *in vitro* (41). On the other hand, some PQs have anticancer activity (42, 43). Although some research has been done on the toxicity of PQs, especially ATX II, it is still unknown how the PQs are biosynthesized (44–46). Perylene quinones are polyketide dimers formed through a phenol-coupling reaction. Other prominent examples for PQs are the phytotoxic cercosporin or elsinochrome, which are produced by *Cercospora* subsp. and *Elsinoë* (47). The gene clusters required for those two toxins have been characterized to some extent (48). There was some evidence that the melanin biosynthesis pathway may be involved in elsinochrome biosynthesis (49, 50). However, later it was shown that the PQ and the DHN melanin synthesis routes are separate pathways in Colletotrichum beticola and in Elsinoe fawcettii (47). Hence, the biosynthesis of PQs is still controversial and not solved, despite the great importance of many of such substances as virulence facilitators in plant-pathogenic fungi as mycotoxins or novel anticancer drugs.

Here, we show that the DHN melanin and the perylene quinone biosynthesis pathways share most of the steps in A. alternata. Our results suggest that PksA produces at least three compounds: YWA1, AT4HN, and T4HN. AT4HN and YWA1 are hydrolyzed to the common intermediate T4HN. However, the pathways are spatially separated and lead to PQ biosynthesis in substrate hyphae, while DHN melanin biosynthesis occurs in aerial hyphae and spores. 1,8-DHN represents the branching point between DHN melanin and ATX biosynthesis. The transcriptional regulator CmrA strictly controls the biosynthesis of ATXs.

## RESULTS

### Polyketide synthase A is involved in DHN melanin and perylene quinone (PQ) biosynthesis.

**FIG 1:**
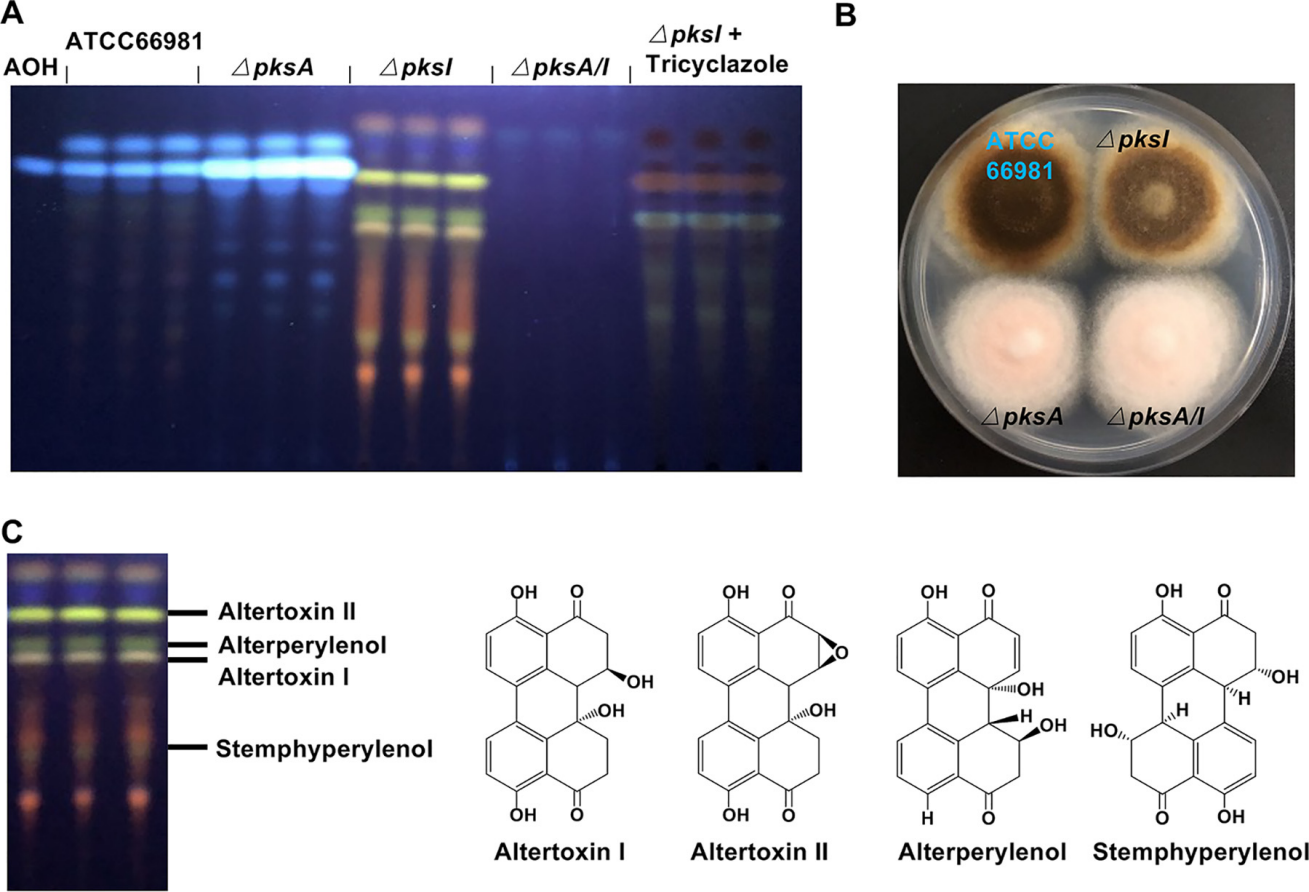
Dual use of polyketide synthase A (PksA) for dihydroxynaphthalene (DHN) melanin and perylene quinone (PQ) biosynthesis. (A) Thin-layer chromatographic (TLC) analysis of extracts from Alternaria alternata strain ATCC 66981 and mutant strains grown on mCDB medium alone or mCDB medium containing 30 mg/mL tricyclazole for 5 days at 28°C. An alternariol (AOH) standard was used for comparison. (B) Growth of the A. alternata ATCC 66981 and mutant strains on mCDB medium for 5 days at 28°C. (C) Assignment of the compounds to the bands obtained in the TLC analysis in panel A. Chemical structures of altertoxin I and II, alterperylenol, and stemphyperylenol.

In A. alternata, the biosynthesis of the polyketide alternariol and several derivatives thereof are encoded by the gene cluster *pksI*. These polyketides are produced in large amounts and easy to detect as prominent blue-fluorescent bands in TLC analyses (30). Although the polyketide synthase, PksI, is sufficient for alternariol production, the biosynthesis of the derivatives requires tailoring enzymes. In a *pksI-*deletion strain deficient of producing alternariol, the blue-fluorescent bands in the TLC analysis disappeared, while four yellow bands became visible (Fig. 1A). In order to study which *pks* gene cluster plays a role in the production of these secondary metabolites (SMs), we deleted other polyketide synthase-encoding genes in the *pksI*-deletion strain using the CRISPR/Cas9 technology (30, 51). The yellow bands disappeared when a functional *pksA* gene was lacking (Fig. 1A). This was unexpected because PksA is required for DHN melanin biosynthesis (33, 34). Colonies of the *pksA/pksI* double-deletion strain appeared indeed pale or pinkish instead of black, like wild type or the *pksI-*deletion strain (Fig. 1B). To further verify that the yellow SMs were produced through the DHN melanin biosynthesis pathway, tricyclazole (30 mg/liter), was used to inhibit the reduction of 1,3,6,8-tetrahydroxynaphthalene (T4HN) to scytalone and 1,3,8-trihydroxynaphthalene (1,3,8-THN) to vermelone, respectively. Addition of the drug prevented accumulation of the yellow SMs (Fig. 1A). To confirm the function of *pksA* in the biosynthesis of melanin and the yellow SMs, the *pksA/pksI* double-deletion strain was recomplemented with the *pksA* wild-type gene. Although melanization was not completely restored (Fig. S1A), the yellow SMs were clearly detected in complemented strains (Fig. S1B). Taken together, we discovered the double use of PksA for producing DHN melanin and several SMs secreted into the medium. This raised questions about the nature of the yellow SMs, the localization of the biosynthesis pathways, and their regulation.

10.1128/mbio.00219-22.1 FIG S1 Melanization and PQ biosynthesis of the *pksA*-deletion strain were rescued through complementation with the *A. alternata pksA* gene. (A) Growth of the wild type (WT), the *pksA*-deletion strain and the complemented strain on mCDB medium for 5 days at 28°C. (B) Thin-layer chromatographic analysis of extracts from WT and the *pksA*-deletion strain and the complemented strain grown on mCDB medium for 5 days at 28°C. Altertoxin II (ATX II) standard was used for comparison. Download FIG S1, TIF file, 1.6 MB. Copyright © 2022 Gao et al. 2022 Gao et al. https://creativecommons.org/licenses/by/4.0/ This content is distributed under the terms of the Creative Commons Attribution 4.0 International license.

The analysis of AOH and ATXs in wild type and the different mutant strains revealed another interesting aspect. In the absence of ATX biosynthesis, more AOH was produced (Δ*pksA* strain), whereas in the absence of the AOH pathway, more ATXs were formed (Δ*pksI* strain). These results suggest that the two pathways compete for the same precursors.

In order to identify the yellow SMs, the respective bands were scratched from the TLC plates, extracted with a mixture of acetonitrile and water, and analyzed with liquid chromatography–high-resolution mass spectrometry (LC-HRMS). Inspection of the LC-HRMS chromatograms revealed the presence of altertoxin II (ATX II), alterperylenol (ALP), and altertoxin I (ATX I), which were identified based on comparison with reference standards, as well as stemphyperylenol (STP), which was annotated based on *m/z*, tandem mass spectrometry (MS/MS) fragmentation behavior, and database search (52) (Fig. 1C) (Table S1). In order to determine the timing of DHN melanin and PQ biosynthesis, the *pksI-*deletion strain was incubated on solid modified Czapek-Dox broth (mCDB) media for 1 to 7 days. Melanization of the *pksI-*deletion strain was clearly detected after 4 days of inoculation (Fig. S2A), whereas the production of PQs was detected after 2 days of inoculation, and after 3 days the amounts of PQs remained constant (Fig. S2B). These results suggest that PQs are produced before A. alternata is melanized and that DHN melanin biosynthesis and PQ biosynthesis may be differentially regulated, although the pathways share at least PksA.

10.1128/mbio.00219-22.2 FIG S2 Timing of DHN-melanin and PQ biosynthesis. (A) Growth of wild type (WT). 5 × 10^4^ spores of WT were spread evenly on the mCDB agar plate and incubated for 1-7 days at 28°C. (B) Thin-layer chromatographic analysis of extracts from WT after 1-7 days of growth on mCDB medium at 28°C. Altertoxin II (ATX II) standard was used for comparison. Download FIG S2, TIF file, 3.0 MB. Copyright © 2022 Gao et al. 2022 Gao et al. https://creativecommons.org/licenses/by/4.0/ This content is distributed under the terms of the Creative Commons Attribution 4.0 International license.

10.1128/mbio.00219-22.7 TABLE S1 Detailed information for the identification of ATX II, ALP and ATX I. Retention time, detection ions, accurate mass of the most abundant ion as well as mass deviation are shown for the reference standards and the respective biological sample from TLC plates. Download Table S1, PDF file, 0.03 MB. Copyright © 2022 Gao et al. 2022 Gao et al. https://creativecommons.org/licenses/by/4.0/ This content is distributed under the terms of the Creative Commons Attribution 4.0 International license.

### Analysis of the DHN melanin gene cluster and synteny in A. alternata.

**FIG 2:**
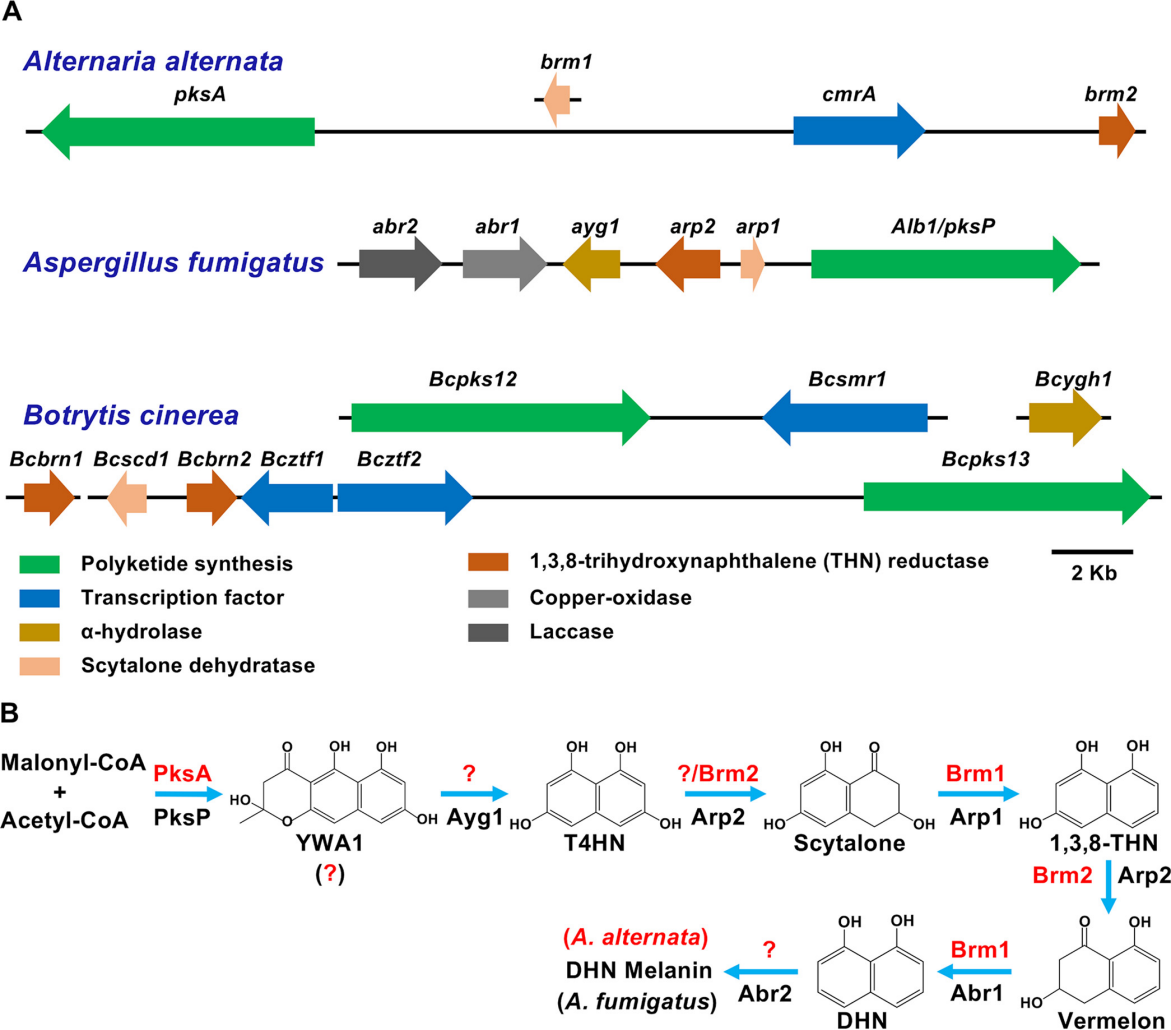
Comparison of DHN melanin biosynthesis pathways in different fungi. (A) Synteny and rearrangements of DHN melanin biosynthetic gene clusters in A. alternata, Aspergillus fumigatus, and Botrytis cinerea. (B) Comparison of the DHN melanin biosynthetic pathway between A. fumigatus and A. alternata. Enzymes involved DHN melanin biosynthesis in A. alternata are shown in red; the orthologues in A. fumigatus are shown in black. YWA1, ,2,5,6,8-tetrahydroxy-2-methyl-2,3-dihydro-4H-naphtho(2,3-b)pyran-4-one; T4HN, 1,3,6,8-tetrahydroxynaphthalene; T3HN, 1,3,8-trihydroxynaphthalene; DHN, 1,8-dihydroxynaphthalene.

Many fungi produce melanized hyphae and/or spores, but the biosynthesis has been studied in detail only in some of them. The genes involved in DHN melanin biosynthesis are generally clustered in the genome. This is, for instance, the case in A. fumigatus, where the polyketide synthase-encoding gene *alb1*/*pksP*, the α-hydrolase-encoding gene *ayg1*, the scytalone dehydratase-encoding gene *arp1*, the 1,3,8-THN reductase-encoding gene *arp2*, the copper oxidase-encoding gene *abr1*, and the laccase-encoding gene *abr2* are found in the same cluster (Fig. 2A) (53). In B. cinerea, a plant-pathogenic fungus, the DHN melanin gene cluster appears to be more complicated. For instance, the α-hydrolase-encoding gene *Bcygh1* is not clustered in the genome. The polyketide synthase-encoding gene *Bcpks12* and the transcription factor-encoding gene *Bcsmr1* are located next to each other and form a small gene cluster. The other genes, the scytalone dehydratase-encoding gene *Bcscd1*, two 1,3,8-THN reductase-encoding genes *Bcbrn1* and *Bcbrn2*, two transcription factor-encoding genes *Bcztf1* and *Bcztf2*, and the polyketide synthase-encoding gene *Bcpks13*, form another cluster in the genome (Fig. 2A). In B. cinerea, the initial step of DHN melanin production is catalyzed by two polyketide synthases BcPks12 (in sclerotia) and BcPks13 (in conidia), and the melanization in sclerotia and conidia is controlled by the transcription factors BcCMR1 and BcZTF1/BcZTF2, respectively (36). In A. alternata, only three genes are combined in a cluster: the polyketide synthase-encoding gene *pksA*, the transcription factor-encoding gene *cmrA*, and the 1,3,8-reductase-encoding gene *brm2*. The scytalone dehydratase-encoding gene *brm1* is located elsewhere in the genome (Fig. 2A). Other genes involved in melanin synthesis were not yet characterized.

Three very different products, namely, the heptaketide YWA1, the hexaketide AT4HN, and the pentaketide T4HN, are synthesized by polyketide synthases from different fungi. For example, YWA1 is synthesized by Alb1/PksP in A. fumigatus and WA in A. nidulans; in B. cinerea, BcPKS12 and BcPKS13 catalyze the formation of T4HN and AT4HN, respectively; and AT4HN is the product of Wangiella dermatitidis WdPks1 (54). YWA1 and AT4HN are hydrolyzed to T4HN before the synthesis of DHN melanin can continue. In A. alternata, the product of PksA is unknown. As a first step to deduce which molecule could be the product, we analyzed the genome for a gene encoding the α-hydrolase. Both the conversion of T4HN to scytalone and the conversion of T3HN to vermelone are catalyzed by Arp2 in A. fumigatus, Brm2 in A. alternata, THR1/THR4 in Bipolaris oryzae, and BcBRN1/BcBRN2 in B. cinerea (Fig. 2B). In addition to Brm2, another THN reductase could be involved in the reduction of T4HN to scytalone in A. alternata like in B. oryzae THR4. A further open question is the conversion of 1,8-DHN to DHN melanin. This step is catalyzed by laccases (multicopper oxidase), namely, Abr2 in A. fumigatus, PbrB in Talaromyces marneffei, and LAC2 in Colletotrichum orbiculare. The characteristics of the genes analyzed in this paper are listed in Table S2.

10.1128/mbio.00219-22.8 TABLE S2 Gene annotation according to https://mycocosm.jgi.doe.gov/Alalte1/Alalte1.home.html. The introns were confirmed by RNAseq. Download Table S2, PDF file, 0.03 MB. Copyright © 2022 Gao et al. 2022 Gao et al. https://creativecommons.org/licenses/by/4.0/ This content is distributed under the terms of the Creative Commons Attribution 4.0 International license.

### T4HN, AT4HN, and YWA1 are products of the polyketide synthase PksA.

To determine the primary product of PksA, *pksA* was heterologously expressed in Aspergillus oryzae using the maltose-inducible promoter *amyB*(*p*). A. oryzae is an excellent host for heterologous expression, because it is heavily used in the food industry, and secondary metabolite production is minimized (55). The integration of the A. alternata *pksA* gene in the genome of A. oryzae was confirmed by PCR (data not shown). A. oryzae strains expressing A. alternata PksA produced a brownish pigment and appeared dark on agar plates. They grew weakly on MPY media. The growth retardation was probably caused by the products of A. alternata PksA. The strains secreted numerous SMs compared to the A. oryzae WT strain. In order to identify the products of A. alternata PksA, extracts were analyzed with LC-HRMS. The resulting chromatograms were inspected for *m/z* traces, putatively representing intact ion molecules of the expected heptaketide YWA1, the hexaketide AT4HN, and the pentaketide T4HN, as well as some related derivatives (Table 1). A. oryzae wild type served as a control. While the corresponding extracted ion chromatogram (EIC) peaks of YWA1 and AT4HN were consistently found in all PksA-expressing A. oryzae strains, T4HN was not detected in any of the replicates (by comparison with the reference standard). Instead, inspection of EICs for closely related compounds indicated the presence of flaviolin, an oxidation product that can spontaneously be formed from T4HN, which was confirmed by its presence in the reference standard (56). Similarly, an analogue oxidation product of AT4HN (sum formula C_12_H_8_O_6_) was also solely detected in the PksA-producing strains extract samples and not found in the wild-type control. Moreover, a putative acetylated scytalone derivative was found.

**TABLE 1:**
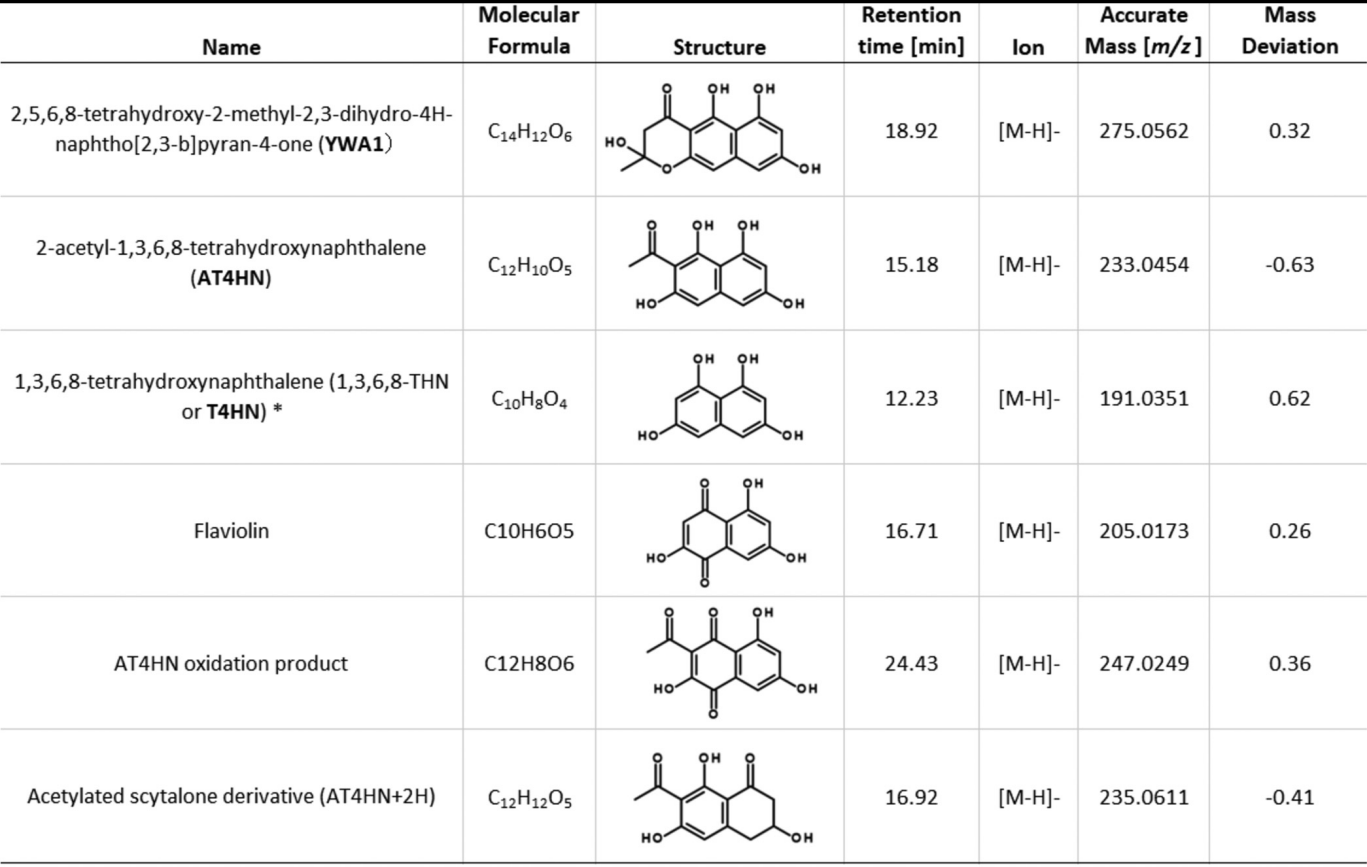
Compound analysis of extracts of the A. oryzae strain expressing A. alternata PksA

*Not found in samples.

To further support PksA product annotation, the presumed PksA products were remeasured by LC-HRMS/MS, and product ion spectra were compared to that of an authentic T4HN standard. Numerous fragments found in the MS/MS spectra of T4HN (*m*/*z* 65, 81, 103, 119, 123, 146, 147, and 149) were also present in the Pks candidates YWA1 and AT4HN, indicating their intact bicyclic phenolic structure. In contrast, the main fragment ions of the putative flaviolin (*m*/*z* 89, 133, 135, and 177) were absent in the T4HN standard, indicating the differences in the core structure. Consistent with these observations, the same fragment ions were also present in the MS/MS spectra of the putative oxidation product of AT4HN, supporting structural similarity of the putative oxidation products. The MS/MS pattern of the acetylated scytalone derivative also shares numerous fragments with the T4HN reference standard (*m/z* 65, 81, 103, 105, 123, 146, and 149), as well as with AT4HN (*m/z* 118, 172, and 190), indicating structural similarity and supporting the annotation.

### Two α-hydrolases, AygA and AygB, are specific for DHN melanin biosynthesis.

Since AT4HN and YWA1 were proven to be the products of A. alternata PksA, an α-hydrolase should be present in A. alternata for further conversion to T4HN. Therefore, we used the protein sequences of Ayg1 from A. fumigatus and WdYg1P from W. dermatitidis to search the A. alternata ATCC 66981 genome database for orthologues genes (https://mycocosm.jgi.doe.gov/Alalte1/Alalte1.home.html). Two candidates, named *aygA* (transcript ID 115293) and *aygB* (transcript ID 105009), were identified and displayed 40.9 and 53.2% identical amino acids compared to A. fumigatus Ayg1 and 59.8 and 44.2% compared to W. dermatitidis WdYg1P, respectively (Fig. S3). The *aygA* gene is comprised of 1,343 bp with one 80-bp intron (confirmed by cDNA sequencing). The *aygB* gene is comprised of 1,212 bp without any intron (confirmed by cDNA sequencing). The derived proteins AygA and AygB consist of 421 and 404 amino acids, respectively.

10.1128/mbio.00219-22.3 FIG S3 The biosynthesis of DHN-melanin and PQ is inhibited by tricyclazole. (A) Growth of wild type (WT) and mutant strains on mCDB medium containing 30 mg/l tricyclazole for 5 days at 28°C. (B) Thin-layer chromatographic analysis of extracts from WT and mutant strains on mCDB medium containing 30 mg/l tricyclazole for 5 days at 28°C. Altertoxin II (ATX II) standard was used for comparison. Download FIG S3, TIF file, 1.7 MB. Copyright © 2022 Gao et al. 2022 Gao et al. https://creativecommons.org/licenses/by/4.0/ This content is distributed under the terms of the Creative Commons Attribution 4.0 International license.

**FIG 3:**
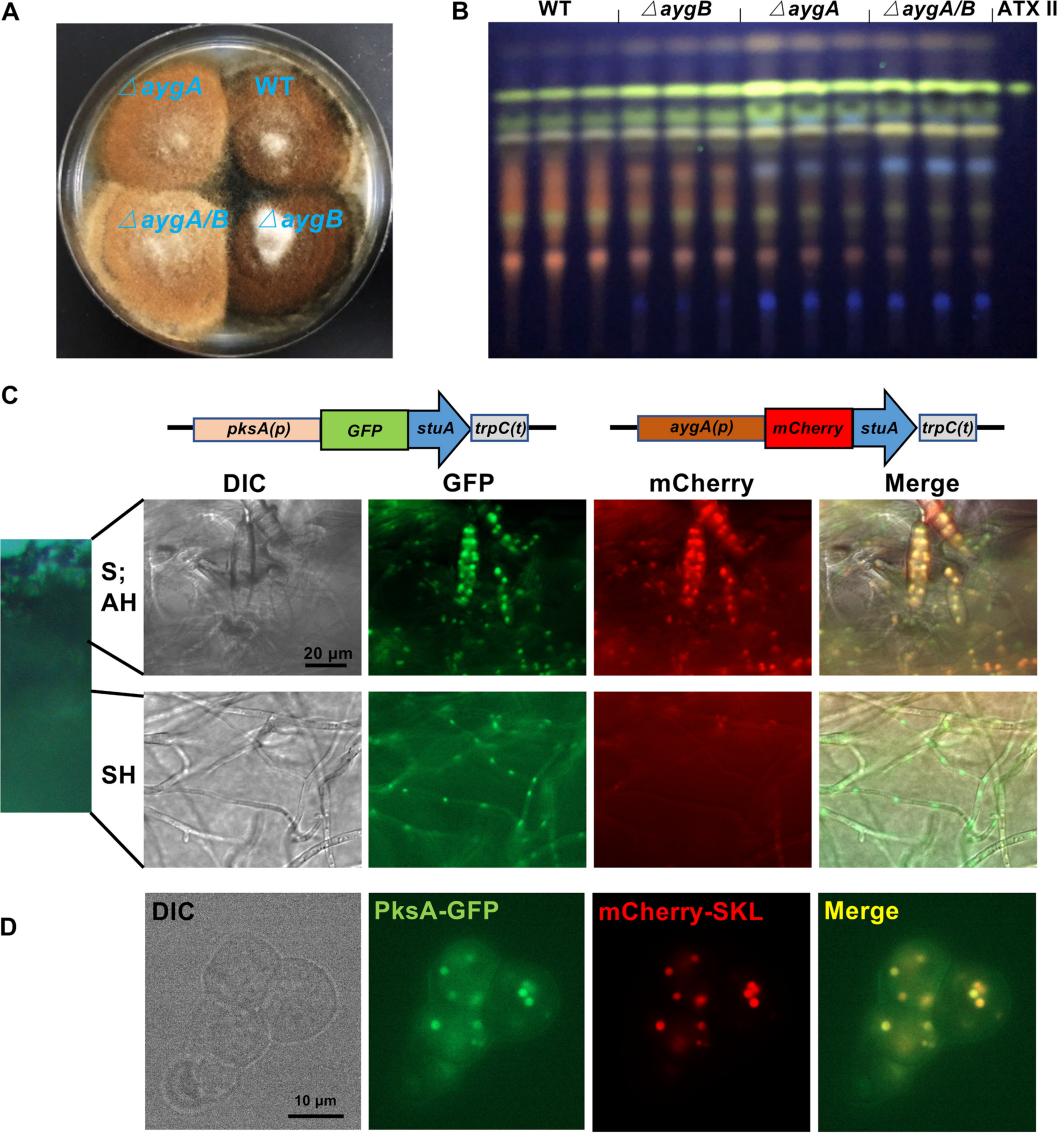
The α-hydrolases AygA and AygB are involved in DHN melanin biosynthesis in spores and aerial hyphae but not in ATX production in substrate hyphae. (A) Growth of wild type (WT) and mutant strains on mCDB medium for 5 days at 28°C. (B) TLC analysis of extracts from WT and mutant strains on mCDB medium for 5 days at 28°C. Altertoxin II (ATX II) standard was used for comparison. (C) Detection of the spatial expression of *pksA* and *aygA* by a promoter-reporter assay. The cassettes of *pksA*(*p*)::*gfp*::*stuA*::*trpC*(*t*) and *aygA*(*p*)::*mCherry*::*stuA*::*trpC*(*t*) were cotransformed into WT protoplasts. A total of 5 × 10^4^ spores from the mutant strain were spread evenly on mCDB agar plates and incubated for 3 days at 28°C. The fluorescent signals were observed in a fluorescence microscope. Bar, 20 μm. (D) Subcellular localization of PksA in spores of strain SJG63 (PksA fused to green fluorescent protein (GFP) and mCherry fused to the peroxisomal targeting sequence SKL). Bar, 10 μm. S, spores; AH, aerial hyphae; SH, substrate hyphae; DIC, differential interference contrast.

To analyze the roles of AygA and AygB in the formation of DHN melanin, both genes were deleted separately and in combination in the *pksI*-deleted strain, using CRISPR/Cas9 technology. The mutant strains were confirmed by PCR and DNA sequencing (Table S3). As our experiments were based on the △*pksI* strain from then on, it was treated as the WT strain. Compared to WT, the Δ*aygA* strain appeared pale brown, whereas no obvious phenotype was observed in the Δ*aygB* strain (Fig. 3A). The double-deletion strain resembled the *aygA*-deletion strain, but even less brown (Fig. 3A). These results indicate that both AygA and AygB may be involved in DHN melanin biosynthesis, with AygA making the greater contribution phenotypically.

10.1128/mbio.00219-22.9 TABLE S3 *Alternaria alternata* and *Aspergillus oryzae* strains used in this study and proof of mutant strains. Download Table S3, PDF file, 1.4 MB. Copyright © 2022 Gao et al. 2022 Gao et al. https://creativecommons.org/licenses/by/4.0/ This content is distributed under the terms of the Creative Commons Attribution 4.0 International license.

10.1128/mbio.00219-22.10 TABLE S4 Oligonucleotides and plasmids used in this study. Download Table S4, PDF file, 0.03 MB. Copyright © 2022 Gao et al. 2022 Gao et al. https://creativecommons.org/licenses/by/4.0/ This content is distributed under the terms of the Creative Commons Attribution 4.0 International license.

Because PksA is also required for PQ biosynthesis, we tested the *aygA/B* deletions strains for their ability to produce PQs and found that neither AygA nor AygB were required (Fig. 3B). However, when these strains were grown on mCDB media containing 30 mg/liter tricyclazole, melanization of these strains was prevented (Fig. S3A), and PQs were not produced (Fig. S3B). These results suggest two routes for DHN biosynthesis: one involving only PksA and T4HN and the other involving PksA plus AygA and AygB to act presumably on YWA1 and AT4HN as intermediates. We next asked whether the different pathways were of any biological meaning and speculated that the expression of the genes could be spatially regulated. It could be that *aygA* and *aygB* are not expressed in substrate hyphae, where T4HN could be the substrate for PQ biosynthesis. If AygA and AygB would be produced in aerial hyphae and spores, the concentration of T4HN should be higher than in substrate hyphae to provide enough precursor for the massive amounts of melanin. To test this hypothesis, we used a promoter-reporter assay based on green fluorescent protein (GFP) or mCherry expression (20). In order to clearly distinguish the specific fluorescent signal from autofluorescence, the fluorescent proteins were targeted to nuclei. The *pksA* promoter was used to express a chimeric protein consisting of the nuclear localization signal (NLS)-containing part of the transcription factor StuA and GFP, and the *aygA* promoter was used for the mCherry reporter (Fig. 3C) (57). Whereas both *pksA* and *aygA* were expressed in spores and arial hyphae, in substrate hyphae, only *pksA* appeared to be expressed. Signals of the *aygA* reporter construct were missing. The same phenomenon was observed when the *aygB* promoter was used for the mCherry reporter (Fig. S4). In addition to the promoter activity analysis, we determined the subcellular localization of PksA. To this end, we inserted GFP before the Stop codon of *pksA* using the CRISPR/Cas9 knock-in strategy. The obtained strain was transformed again to introduce a peroxisomal marker plasmid. The green and red fluorescent signals clearly overlapped, indicating peroxisomal localization of PksA (Fig. 3D). This is in agreement with the localization of Pks12/13 in B. cinerea and different from the localization of Alb1 in A. fumigatus (58, 59).

10.1128/mbio.00219-22.4 FIG S4 Analysis of the spatial expression of *pksA* and *aygB* by a promoter-reporter assay. The cassettes of *pksA(p)::gfp::stuA::trpC(t)* and *aygB(p)::mCherry::stuA::trpC(t)* were co-transformed into wild type (WT) protoplasts. 5 × 10^4^ spores of the formed mutant strain were spread evenly on the mCDB agar plate and incubated for 3 days at 28°C. The fluorescent signals were observed using fluorescence microscopy. Scale bar: 20 μm. S: spores; AH: aerial hyphae; SH: substrate hyphae. Download FIG S4, TIF file, 1.9 MB. Copyright © 2022 Gao et al. 2022 Gao et al. https://creativecommons.org/licenses/by/4.0/ This content is distributed under the terms of the Creative Commons Attribution 4.0 International license.

To prove spatial production of PQs and melanin, WT was incubated on mCDB medium for 3 days. Then, spores and aerial hyphae were collected using a razor blade carefully into one 1.5-mL microtube, and the remaining substrate hyphae together with agar were collected into another 1.5-mL microtube, for the further TLC analysis (Fig. S5A). As we expected, PQs were not detected in spores and aerial hyphae, while they were clearly present in substrate hyphae (Fig. S5B). We also analyzed the spatial production of alternariol. For this, we grew the colonies for 5 days and detected AOH mainly in substrate hyphae. Some AOH signal was also detected in spores and aerial hyphae, although this is more likely a contamination from substrate hyphae. In comparison to ATXs much more AOH is produced, and the separation of the different mycelial types is not perfect. In submerged culture, ATXs appeared earlier than AOH, suggesting that the two pathways likely do not compete for the same substrate.

10.1128/mbio.00219-22.5 FIG S5 PQs are synthesized in substrate hyphae and not in spores and aerial hyphae. (A) Separation of spores/aerial hyphae and substrate hyphae. 10^4^ of spores of WT were spotted on a mCDB agar plate and incubated for 3 days at 28°C. Spores and aerial hyphae from the whole colony were collected into a 1.5 ml micro tube using a razor blade. Substrate hyphae together with mCDB agar were collected into another 1.5 ml micro tube. (B) Thin-layer chromatographic analysis of extracts from spores/aerial hyphae and substrate hyphae from the Δ*pksI* and the wild-type strain. Altertoxin II (ATX II) standard was used for comparison. S: spores; AH: aerial hyphae; SH: substrate hyphae. (C) TLC analysis of ATXs and AOH of extracts from WT grown in submerged culture. Download FIG S5, TIF file, 2.0 MB. Copyright © 2022 Gao et al. 2022 Gao et al. https://creativecommons.org/licenses/by/4.0/ This content is distributed under the terms of the Creative Commons Attribution 4.0 International license.

These results suggest that T4HN is likely to be the substrate for PQ biosynthesis in A. alternata substrate hyphae. *aygA* and *aygB* expression in aerial hyphae and spores favor DHN melanin production.

### A new T4HN reductase, Brm3, is required for DHN melanin and PQ biosynthesis.

**FIG 4:**
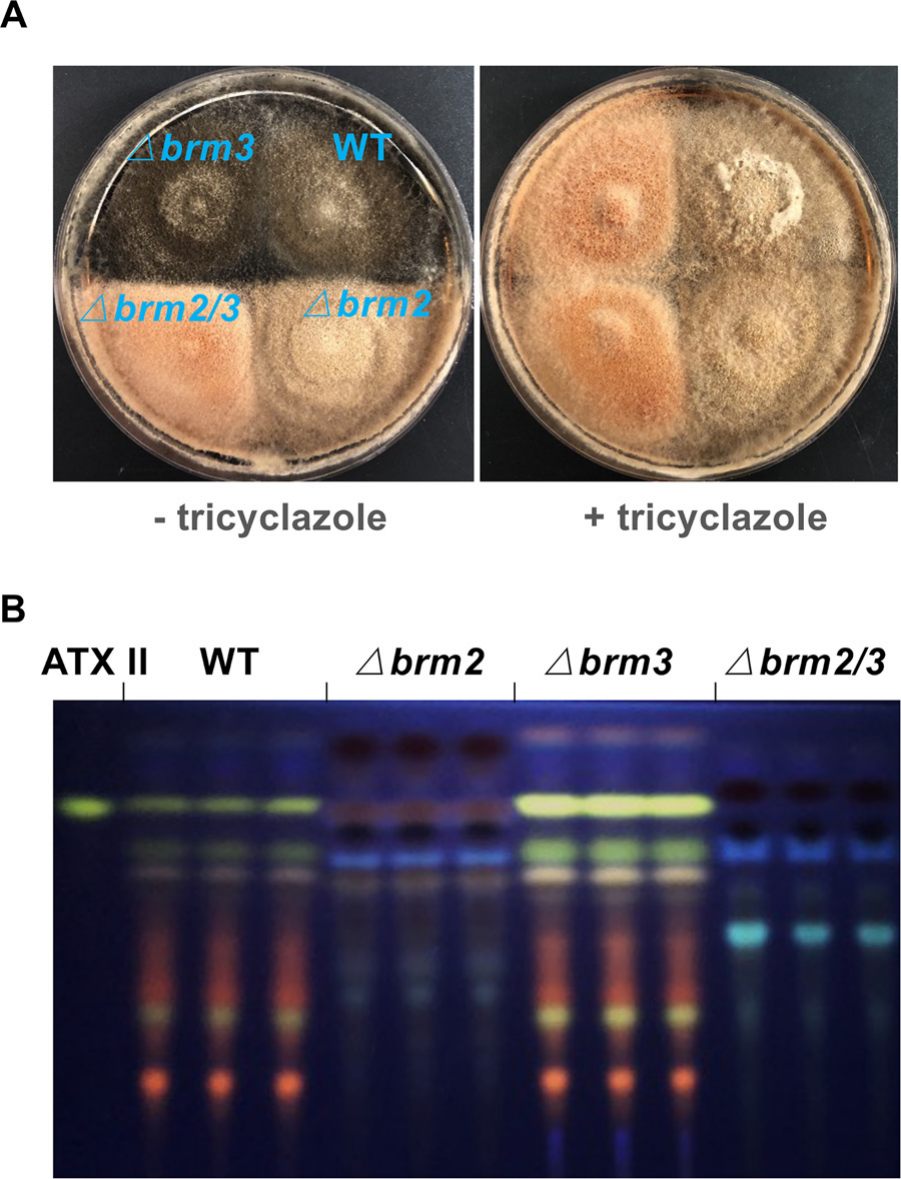
Two T4HN reductases, Brm2 and Brm3, are involved in DHN melanin and ATX biosynthesis. (A) Growth of wild type (WT) and mutant strains on mCDB medium or mCDB medium containing 30 μg/mL tricyclazole at 28°C for 14 days. (B) TLC analysis of extracts from WT and mutant strains grown on mCDB medium for 5 days at 28°C. The altertoxin II (ATX II) standard was used for comparison.

Because there was evidence for two T4HN reductases in B. oryzae, we searched the A. alternata ATCC 66981 genome for a B. oryzae T4HR1 orthologue and found a candidate *brm3* (transcript ID 112254). Brm3 from A. alternata displays 49.8, 48, and 90.4% identical amino acids compared to A. fumigatus Arp2, A. alternata Brm2, and B. oryzae T4HR1, respectively. The *brm3* gene is comprised of 807 bp with no predicted intron (confirmed by cDNA sequencing). The derived protein Brm3 is comprised of 269 amino acids. To explore the role of Brm3 in melanin and PQs biosynthesis, *brm3* was deleted in WT and in a *brm2*-deletion strain. The Δ*brm2*, *Δbrm3*, and *Δbrm2/3* strains were confirmed by PCR and DNA sequencing (Table S3). The *brm3-*deletion strain still appeared black on mCDB medium, whereas the Δ*brm2* strain looked brownish, and the *brm2/3*-double-deletion strain was even less pigmented. The double-deletion strain appeared light orange, probably due to another pigment that normally is hidden by melanin or because another pigment is formed from the accumulating intermediates. Hence, Brm3 also plays a role in melanin formation. However, the △*brm3* and △*brm2/3* strains displayed the same phenotype (orange colonies), and the △*brm2* and WT strains had the same phenotype (brownish colonies) on mCDB media containing 30 mg/liter tricyclazole after 14 days of incubation (Fig. 4A). These results suggest that both Brm2 and Brm3 are isoenzymes reducing T4HN to scytalone, and the Brm2 activity could be inhibited by tricyclazole, but Brm3 activity could not. All mutant strains were also tested for PQ biosynthesis. Whereas the *Δbrm2* and the Δ*brm2/3-*deletion strains did not produce PQs, the *Δbrm3-*deletion strain produced more PQs (Fig. 4B).

### Scytalone dehydratase, Brm1, is involved in PQ biosynthesis.

**FIG 5:**
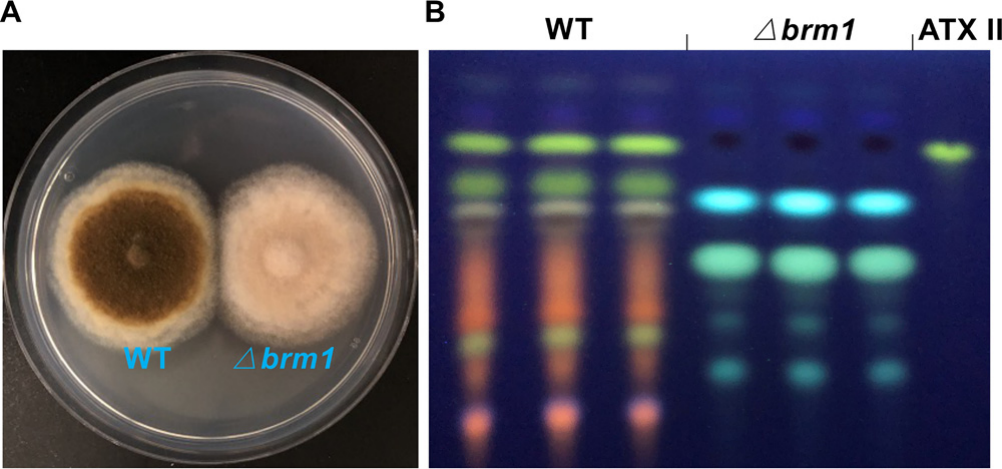
Scytalone dehydratase Brm1 is required for DHN melanin and ATX production. (A) Growth of the wild type (WT) and the *brm1*-deletion strain on mCDB medium for 5 days at 28°C. (B) TLC analysis of extracts from WT and mutant strains grown on mCDB medium for 5 days at 28°C. The altertoxin II (ATX II) standard was used for comparison.

To explore whether Brm1 is required for PQ production, *brm1* was deleted in the WT strain. The Δ*brm1* strain was confirmed by PCR and DNA sequencing (Table S3). The Δ*brm1* strain appeared slightly brownish, suggesting reduced DHN melanin production compared to WT (Fig. 5A). The Δ*brm1* strain produced several other SMs instead of PQs (Fig. 5B).

### 1,8-DHN as last common intermediate of PQ and DHN melanin biosynthesis.

**FIG 6:**
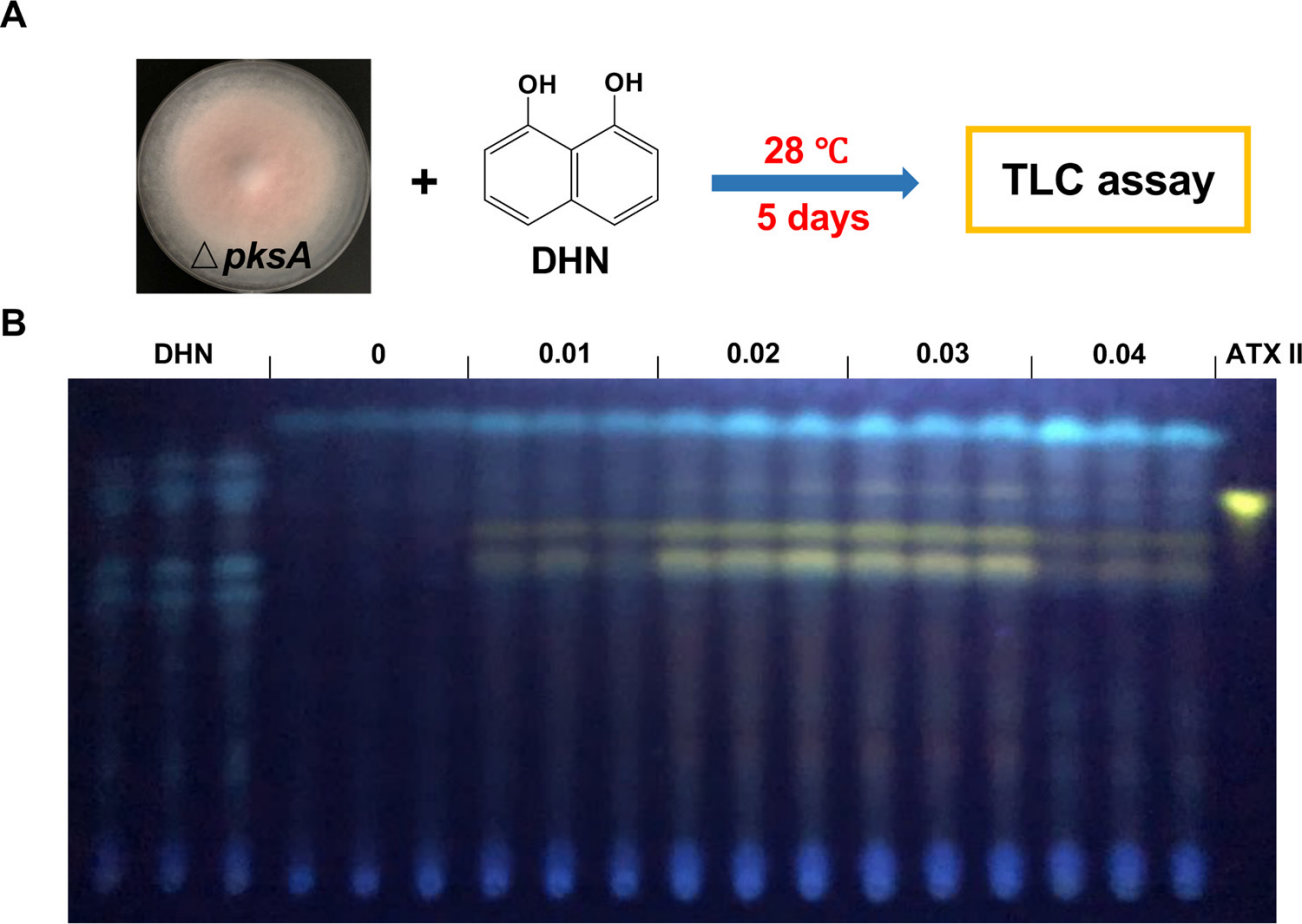
1,8-DHN is the intermediate used for synthesizing ATXs. (A) Scheme of the feeding experiment. The *pksA*-deletion strain was grown on mCDB media containing different amounts of 1,8-DHN (0, 0.01, 0.02, 0.03, and 0.04 mg/mL) for 5 days at 28°C, respectively. mCDB medium containing 0.02 mg/mL 1,8-DHN without inoculation was used as a negative control. (B) Extracts prepared from the different assays were analyzed through TLC analysis. The altertoxin II (ATX II) standard was used for comparison. 1,8-DHN, 1,8-dihydroxynaphthalene.

The absence of both Brm1 and Brm2 interrupted the PQ biosynthesis, and Brm1 could catalyze the dehydration of scytalone and vermelon to 1,3,8-THN and 1,8-DHN, respectively (Fig. 2B). Therefore, we asked whether vermelon or 1,8-DHN is the intermediate used for PQ formation in the DHN melanin biosynthesis pathway. To study whether 1,8-DHN (vermelon could not be obtained commercially) could be utilized to produce PQs, different amounts of 1,8-DHN (0 to 0.04 mg/mL) were added to mCDB media and inoculated with the Δ*pksA*-deletion strain (Fig. 6A). mCDB media containing 0.02 mg/mL 1,8-DHN without inoculating the *pksA*-deletion strain was used as control. After 5 days of incubation, ATX II, ATX I, and ALP were clearly detected, and their amounts increased with increasing concentrations of 1,8-DHN (0.01 to 0.03 mg/mL) (Fig. 6B). At a concentration of 0.04 mg/mL 1,8-DHN, the production of the secondary metabolites was reduced, probably because of toxic side effects of 1,8-DHN. The Δ*pksA*-deletion strain was highly melanized after 5 days when grown in liquid medium in shaking cultures containing 0.02 mg/mL 1,8-DHN (Fig. S6). These results show that 1,8-DHN represents the branching point between the melanin and the PQs biosynthesis pathways.

10.1128/mbio.00219-22.6 FIG S6 Melanization of the *pksA*-deletion strain was rescued through feeding with 1,8-DHN. 10^5^ of spores of the *pksA*-deletion strain were incubated in liquid mCDB medium or mCDB medium containing 0.02 mg/ml 1,8-DHN in a flask for 5 days at 28°C, 180 rpm. 1,8-DHN, 1,8-dihydroxynaphthalene. Download FIG S6, TIF file, 1.8 MB. Copyright © 2022 Gao et al. 2022 Gao et al. https://creativecommons.org/licenses/by/4.0/ This content is distributed under the terms of the Creative Commons Attribution 4.0 International license.

### From seven laccases, four are involved in melanin production but not in PQ biosynthesis.

**FIG 7:**
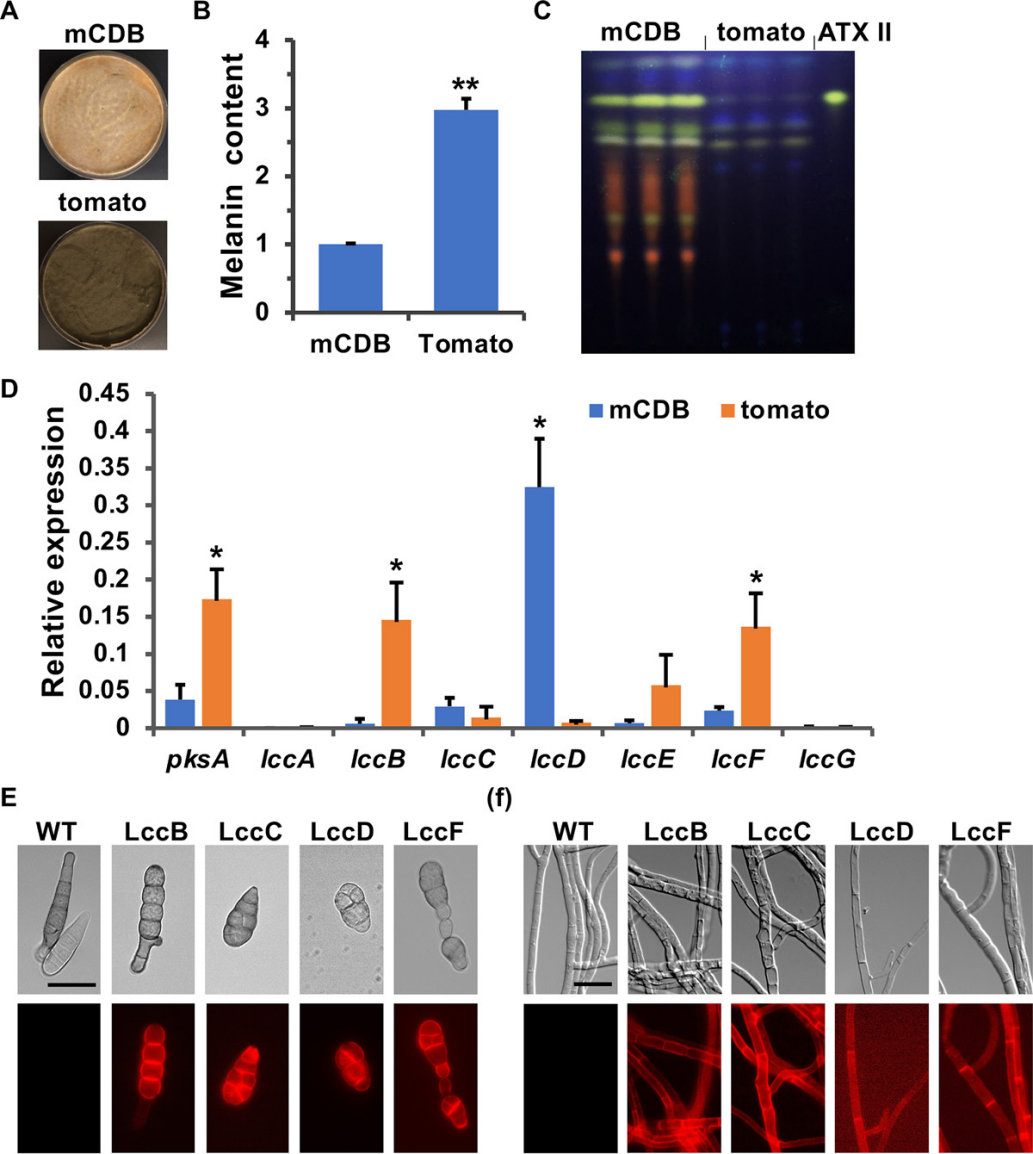
Four laccases, LccB, LccC, LccD, and LccF, as candidates for melanin production. (A) Growth of wild type (WT) on mCDB medium or mCDB medium containing tomato puree (400 g/liter). A total of 5 × 10^4^ spores were spread evenly on the agar surface and incubated for 5 days at 28°C. (B) Quantitative analysis of the melanin content of WT grown on mCDB medium or mCDB medium containing tomato puree (400 g/liter) for 5 days at 28°C. (C) TLC analysis of extracts from WT grown on mCDB medium or mCDB medium containing tomato puree (400 g/liter) for 5 days at 28°C. The altertoxin II (ATX II) standard was used for comparison. (D) Expression analysis of laccase-encoding genes (*lccA* to *lccG*) in WT strain grown on mCDB medium or mCDB medium containing tomato puree (400 g/liter) at 28°C for 5 days. RNA was isolated, and transcript levels were determined by quantitative real-time PCR using specific primers for the genes analyzed. *h2b* was used as the endogenous control for gene expression analysis. (E) Subcellular localization of LccB, LccC, LccD, and LccF in spores. Bar, 10 μm. (F) Subcellular localization of LccB, LccC, LccD, and LccF in aerial hypha. Bar, 20 μm. Error bars represent the standard deviation of three biological replicates. Statistical analysis was performed with Student’s test, *, *P* ≤ 0.05; **, *P* ≤ 0.01. Tomato, mCDB medium containing tomato puree (400 g/liter).

Laccases are considered key enzymes to polymerize 1,8-DHN to melanin in fungi. Furthermore, laccases mediating phenol-coupling reactions are involved in the production of mycotoxins, such as cercosporin in C. beticola, viriditoxin in Aspergillus viridinutans and Paecilomyces variotiin, and sporandol in Chrysosporium merdarium (48). Therefore, we tested whether a laccase could play a role in PQ and/or melanin production. Seven putative laccase-encoding genes (named *lccA* to *lccG*) were identified in the genome of A. alternata using amino acid sequences of known laccases from different fungi. In order to get first indications regarding which of the seven laccases could be involved in melanin and/or PQ biosynthesis, growth and gene expression analyses were performed. A. alternata produced more melanin but less PQs when grown on mCDB media containing tomato puree (400 g/liter) (Fig. 7A to C), the expression of *lccB*, *lccE*, and *lccF* was significantly upregulated (23.8, 8.6, and 5.8 times higher, respectively), and the expression of *lccD* was highly downregulated (44.1 times lower). The expression of *lccA* and *lccG* was barely detectable under both conditions, and no obvious difference was observed for the expression of *lccC* (Fig. 7D). Hence, we speculated that LccB, LccE, and LccF were good candidates for DHN melanin biosynthesis, and LccD could contribute to the formation of PQs.

Next, we analyzed putative domains in the laccases, because laccases involved in melanin formation should be secreted, like Abr2 in A. fumigatus (60). With the exception of LccE, the other six laccases (LccA, LccB, LccC, LccD, LccF, and LccG) have a potential signal peptide at the N terminus. LccA and LccG have a transmembrane domain close to their C terminus. Comparison with laccases from different fungi suggested that the laccase involved in DHN melanin biosynthesis should contain a signal peptide but not a transmembrane domain. Therefore, LccB, LccC, LccD, and LccE are good candidates to be involved in the formation of DHN melanin. The functionality of the signal peptide for all four laccases was tested with mCherry-tagged enzymes (Fig. 7E and F). All of them labeled the contour of the spores and hyphae, suggesting secretion or cell wall localization (58).

**FIG 8:**
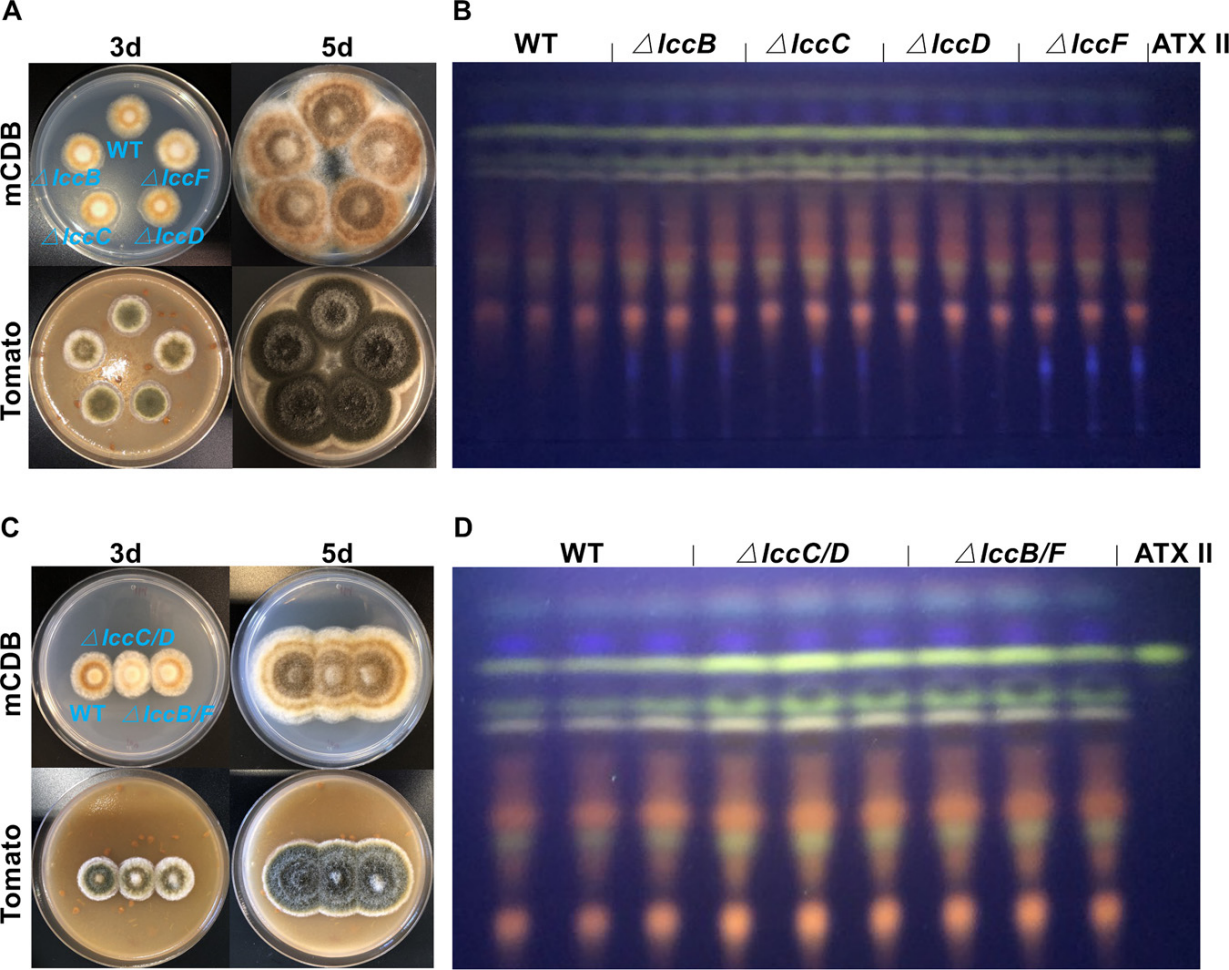
Four laccases, LccB, LccC, LccD, and LccF, are involved in melanin production but not in ATXs biosynthesis. (A) Growth of wild type (WT), Δ*lccB*, Δ*lccC*, Δ*lccD*, and Δ*lccF* strains on mCDB medium or mCDB medium containing tomato puree (400 g/liter) for 3 or 5 days at 28°C. (B) TLC analysis of extracts from WT, Δ*lccB*, Δ*lccC*, Δ*lccD*, and Δ*lccF* strains grown on mCDB medium for 5 days at 28°C. (C) Growth of WT, Δ*lccC*/*D*, and Δ*lccB*/*F* strains on mCDB medium or mCDB medium containing tomato puree (400 g/liter) for 3 or 5 days at 28°C. (D) TLC analysis of extracts from WT, Δ*lccC*/*D*, and Δ*lccB*/*F* strains grown on mCDB medium for 5 days at 28°C. The altertoxin II (ATX II) standard was used for comparison.

To assign functions for these four laccases, *lccB*, *lccC*, *lccD*, and *lccF* or combinations thereof were deleted in WT. Compared to WT, the melanization of *lccB*-, *lccC*-, *lccD*-, and *lccF*-deleted strains was not significantly influenced when grown on mCDB media for 3 and 5 days. However, the melanization of *lccB-* and *lccF-d*eleted strains was highly reduced when grown on mCDB media containing tomato puree (400 g/liter) for 3 days. And after 5 days of incubation, the melanization of both mutants was not influenced (Fig. 8A). PQ biosynthesis appeared also to be unaffected (Fig. 8B). In addition, the melanization of *lccC*/*D*- and *lccB*/*F*-deletion strains was highly reduced when grown on mCDB or mCDB medium containing tomato puree (400 g/liter) for 3 days, and the absence of LccC/D and LccB/F decreased the melanization level after 5 days of incubation (Fig. 8C). In contrast, the production of PQs in both mutant strains appeared quite normal (Fig. 8D).

These results suggest that LccB, LccC, LccD, and LccF are involved in the biosynthesis of DHN melanin to some extent. However, they seem to have some redundant functions, and the lack of some laccases could be substituted for by other laccases. However, none of the investigated laccases appears to be involved in PQs biosynthesis.

### Regulation of the DHN melanin and PQ biosynthesis pathway.

**FIG 9:**
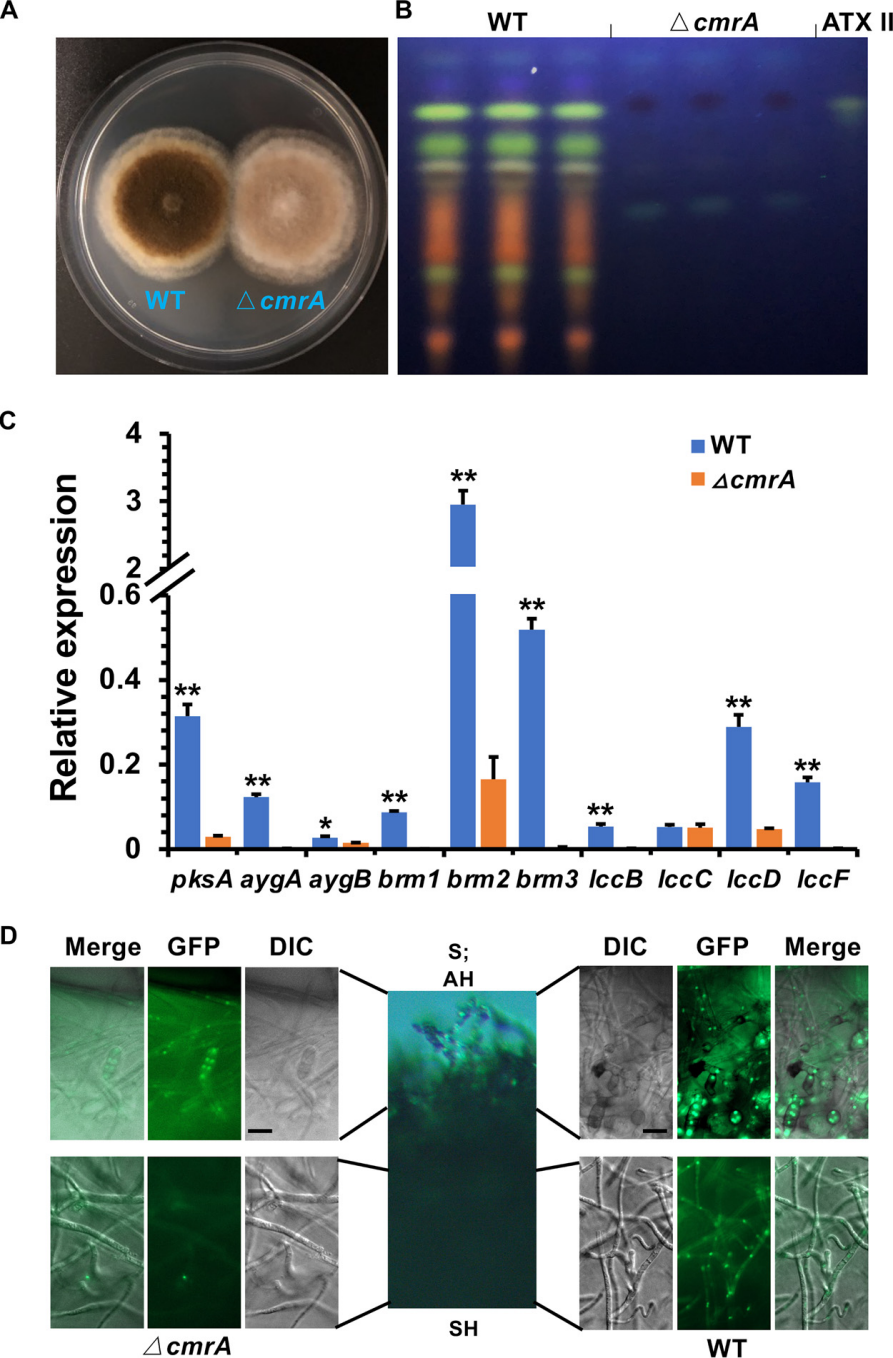
The transcription factor CmrA strictly controls the production of PQs in substrate hyphae. (A) Growth of the wild type (WT) and *cmrA*-deletion strain on mCDB medium for 5 days at 28°C. (B) TLC analysis of extracts from WT and *cmrA*-deleted strain grown on mCDB medium for 5 days at 28°C. The altertoxin II (ATX II) standard was used for comparison. (C) Expression analysis of genes involved in 1,8-DHN melanin and/or PQs biosynthesis in WT and *cmrA*-deleted strains grown on mCDB medium for 5 days at 28°C. The *h2b* gene was used as the endogenous control for gene expression analysis. Error bars represent the standard deviation of three biological replicates. Statistical analysis was performed with Student’s test, *, *P* ≤ 0.05; **, *P* ≤ 0.01. (D) Detection of the spatial expression of *pksA* by a promoter-reporter assay. The cassette of *pksA*(*p*)::*GFP*::*stuA*::*trpC*(*t*) was transformed into WT and the *cmrA*-deletion strain, respectively. A total of 5 × 10^4^ spores of the transgenic strains were spread evenly on the mCDB agar plate and incubated for 3 days at 28°C. The fluorescent signals were observed using fluorescence microscopy. Bar, 20 μm. S, spores; AH, aerial hyphae; SH, substrate hyphae.

In this study, we characterized 10 genes required for DHN melanin biosynthesis, from which PksA, Brm1, Brm2, and Brm3 are also required for PQ biosynthesis. Next, we tested whether these genes were all under the control of the transcription factor CmrA, whose encoding gene is located close to the *pksA* gene. *cmrA* was deleted in WT, and *cmrA* deletion was confirmed by PCR and DNA sequencing (Table S3). The *ΔcmrA* strain appeared brownish (Fig. 9A), not like the pale or pinkish *ΔpksA* strain (Fig. 1A), and did not produce PQs (Fig. 9B). To this end, we quantified the transcript levels of all genes involved in PQs and/or melanin biosynthesis by quantitative real-time PCR. With the exception of *lccC*, the other nine genes were all less expressed in the *cmrA-*deletion strain compared to WT (Fig. 9C). The expression of *aygA*, *brm1*, *brm3*, *lccB*, and *lccF* was strictly dependent on CmrA, whereas the expression of *pksA*, *aygB*, *brm2*, and *lccD* were only partially dependent on the transcription factor. Interestingly, deletion of *cmrA* did not cause an albino phenotype but only a reduction of pigmentation, suggesting that additional cues or transcription factors are involved. To get a spatial rather than only a time resolution, we analyzed the expression of *pksA* in the Δ*cmrA* strain using the promoter-reporter assay as described above. A cassette of *pksA*(*p*)::*stuA*::*GFP*::*trpC*(*t*) was transformed into the △*cmrA* and WT strains, respectively. Fluorescent signals were detected in nuclei of spores and aerial hyphae in both WT and the *cmrA*-deletion strain, but only in nuclei of substrate hyphae in WT (Fig. 9D). Taken together, these results suggest that the production of PQs is strictly controlled by the transcription factor CmrA, whereas DHN melanin biosynthesis can also occur in the absence of CmrA to some extent.

## DISCUSSION

**FIG 10:**
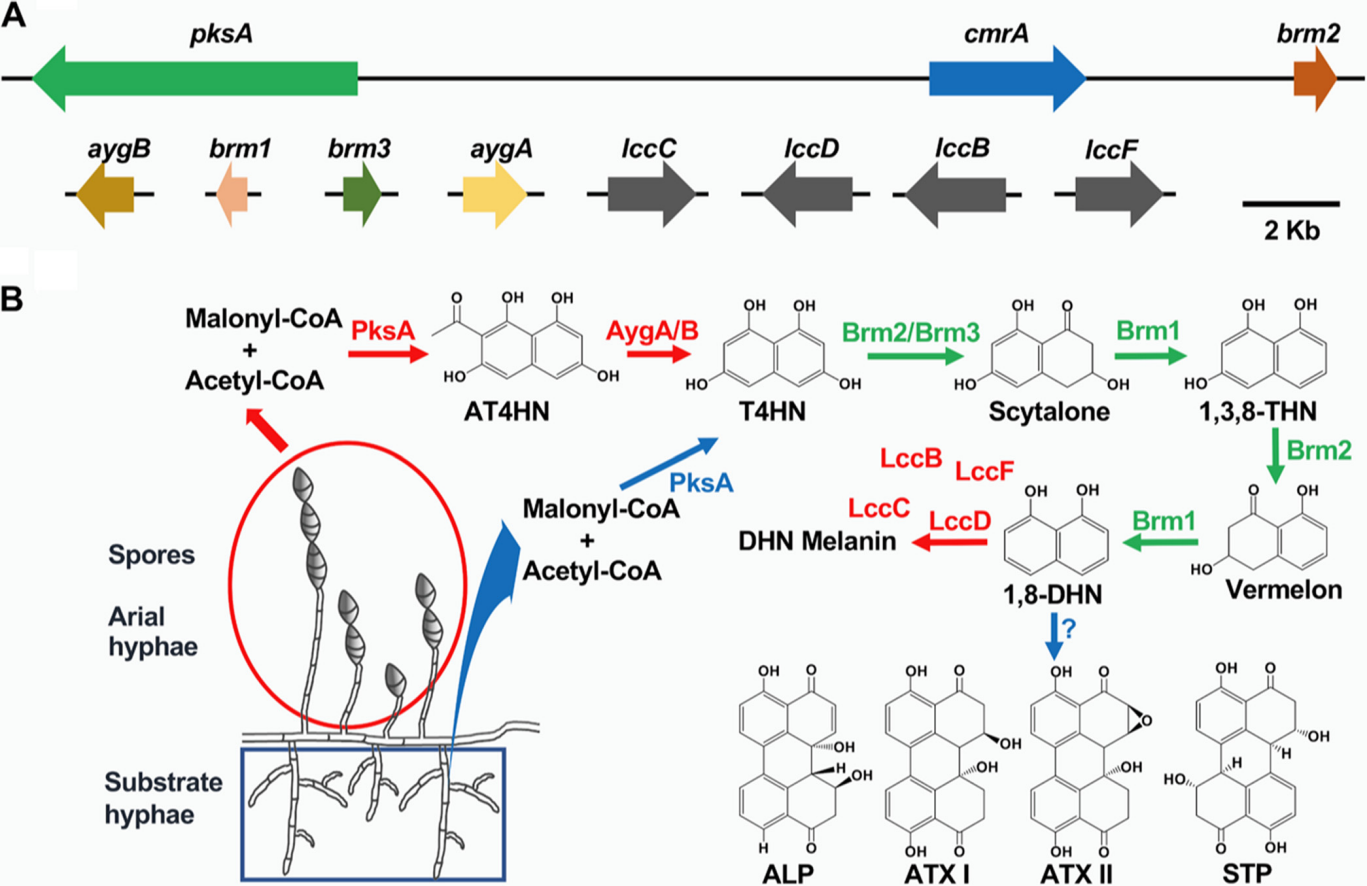
Scheme of the DHN melanin and PQ biosynthetic pathways in A. alternata. (A) Synteny and rearrangements of the DHN melanin and PQs biosynthetic gene cluster in the genome in A. alternata. Except for three genes, namely, the polyketide synthase-encoding gene *pksA*, the 1,3,8-trihydroxynaphthalene (THN) reductase-encoding gene *brm2*, and the transcription factor-encoding gene *cmrA* clustered in the genome, other genes involved in DHN melanin and/or PQs biosynthesis, such as α-hydrolase-encoding genes *aygA* and *aygB*, scytalone dehydratase-encoding gene *brm1*, 1,3,6,8-tetrahydroxynaphthalen (T4HN) reductase-encoding gene *brm3*, and laccase-encoding genes *lccB*, *lccC*, *lccD*, and *lccF*, are scattered. (B) Spatial separation of DHN melanin and PQs biosynthesis in A. alternata. DHN melanin is synthesized in aerial hyphae and spores, whereas the production of PQs occurs in substrate hyphae. α-Hydrolase AygA and AygB are involved in DHN melanin but not PQs biosynthesis. The dimerizing enzyme forming PQs from 1,8-DHN remains to be discovered. Enzymes involved in DHN melanin biosynthesis are shown in red; enzymes involved PQs biosynthesis are shown in blue; enzymes shared by DHN melanin and PQs biosynthesis are shown in green. ATX, altertoxin; ALP, alterperylenol; STP, stemphyperlenol.

A large proportion of pharmaceuticals is based on natural secondary metabolites from plants, fungi, and bacteria. The discovery that many secondary metabolite gene clusters in fungal genomes are expressed only under certain environmental conditions and that their products remained undiscovered so far stimulates researchers to reopen that treasure box and find new, urgently needed antibiotics or other pharmaceuticals (10, 61–63). Strategies include activation of endogenous gene clusters by imitation of natural conditions or by heterologous expression of putative gene clusters (12, 64–66). In addition, detailed analyses of secondary metabolite gene clusters will help to understand the complexity of biosynthetic pathways accounting for the great variety of different organic molecules. For a long time, the aflatoxin gene cluster dominated our understanding of gene organization and regulation of secondary metabolite gene clusters. All genes are found within the cluster, including a regulator for the coordinated activation of all cluster genes. Here, we found that DHN melanin and PQ biosynthesis share mostly the same pathway but that the two pathways are spatially regulated (Fig. 10). Whereas melanin is produced in older aerial hyphae and in spores, PQs are produced in substrate hyphae. The initial steps of the biosynthesis are identical and are catalyzed by the polyketide synthase A PksA. However, in aerial hyphae, the primary products appear to be YWA1 and AT4HN, which are further converted to T4HN by AygA and AygB. In substrate hyphae, PksA probably synthesizes T4HN as the only product and/or cannot use YWA1 or AT4HN to generate T4HN. This was unexpected because often polyketide synthases, producing DHN melanin, appear to have different enzymatic properties and produce T4HN, YWA1, or AT4HN (36, 37). The A. alternata PKS shows obviously some promiscuity and produces the pentaketide T4HN and the hexa- and the heptaketides, YWA1 and AT4HN. A similar promiscuity has been described in the terrein biosynthesis pathway, where 4-hydroxy-6-methylpyranone, orsellinic acid, and 6,7-dihydroxymellein were identified (67). However, it was speculated that only one of the products would be further converted. In A. alternata, it is likely that indeed all three products are further processed, given that the expression of the hydrolase enzymes was found in aerial hyphae and spores. Therefore, one can speculate that in aerial hyphae and in spores, the amount of T4HN is higher than in substrate hyphae. The additional conversion of YWA1 and AT4HN to T4HN in aerial hyphae and spores could boost the biosynthesis of the DHN melanin intermediates and push the biosynthesis toward melanin. In substrate hyphae, the lower concentration of T4HN could support PQ biosynthesis but could be too low for significant melanin formation. Another possibility for the observed variation of the PksA products could be the use of YWA1 and/or AT4HN as the substrate for other unknown compounds produced in substrate hyphae. Finally, there is the possibility that not all three products are produced simultaneously but that PksA produces T4HN in substrate hyphae and YWA1 and AT4HN in aerial hyphae and spores. The change of the PksA specificity could be achieved by structural changes of PksA, for instance through specific interacting proteins. It also remains to be shown which are the direct products of PksA in A. alternata, since the identified products were generated in A. oryzae as heterologous host. The fact that in a similar approach with the Colletotrichum lagenarium PKS mainly T4HN was produced, as well as minor amounts of the tetraketide orsellinic acid and the pentaketide isocoumarin, suggests that the occurrence of the hexa- and the heptaketide in our expression experiment is specific for A. alternata PksA and not due to further conversions by A. oryzae enzymes (56).

Another interesting aspect of PQ biosynthesis is the dimerizing enzyme. Four different enzyme classes are able to perform such phenol coupling reactions, cytochrome P450, flavin-dependent monooxygenases, peroxidases, or laccases (48, 68, 69). We deleted several laccases from the genome and created some double-deletion strains. However, they were all able to produce PQs. Although it could be that yet other laccases could perform the same reaction, it appears currently unlikely that laccases play an important role in PQ biosynthesis in A. alternata. For the biosynthesis of the biaryl sporandol from C. merdarium, a combination of a laccase with a fasciclin domain-containing enzyme was required (69). Such a fasciclin along with a laccase was also found in the cercosporin biosynthesis gene cluster, and it was speculated that it is required for cercosporin formation (48). In A. alternata, we found a conserved fasciclin protein (35.2% identical amino acids compared to C. beticola), but deletion did not affect PQ formation. If reverse-genetic approaches do not help to discover the dimerizing enzyme, other methods such as enzyme purification or forward-genetic approaches will be required. We found that CmrA strictly controls PQ biosynthesis and only to some extent DHN melanin biosynthesis. This may be explained by strict regulation of *pksA* and/or the dimerizing enzyme-encoding gene by CmrA. However, another possibility is that not a single enzyme is able to perform the coupling reaction but that several different enzymes fulfill that function. The activity could even be a side reaction of an enzyme, given that laccases or cytochrome P450 oxidases can be rather unspecific. If this were the case, of course, the lack of a single enzyme would not affect the ability to produce PQs. Considering this aspect fosters the hypothesis that other fungi that produce melanin with DHN as an intermediate may convert some of the intermediates (DHN or scytalone) to PQs or related compounds. Hence, the production of PQs could be much more common in the fungal kingdom than so far anticipated.

The molecular analysis of PQ and melanin biosynthesis revealed spatial separation of their production and suggests that certain physiological conditions of the mycelium favor toxin biosynthesis, whereas others prevent toxin formation. The next step should be the determination of the exact parameters that distinguish substrate hyphae and arial hyphae and spores. This may help to develop schemes for food and feed processing and/or storage to minimize PQ toxin production until consumption.

## MATERIALS AND METHODS

### Strains, plasmids, and culture conditions.

A. alternata and A. oryzae strains are listed in Table S3 which also contains the confirmation of the mutant strains. A. alternata strains were grown on mCDB medium (4% glucose, 0.1% yeast extract, 0.1% NaNO_3_, 0,025% NH_4_Cl, 0.1% KH_2_PO_4_, 0.025% KCl, 0.025% NaCl, 0.05% MgSO_4_ 7×H_2_O, 0.001% FeSO_4_ 7×H_2_O, 0.001% ZnSO_4_, and 1.5% agar) alone or on mCDB medium containing tomato puree (400 g/liter) at 28°C. A. oryzae strain NSAR1 was grown on mCD medium (1.5% glucose, 0.4% yeast extract, 0.1% KH_2_PO_4_, 0.05% MgSO_4_ 7×H_2_O, and 1.5% agar) at 28°C. For metabolite expression, A. oryzae strains were incubated on MPY medium (2% maltose, 1% polypeptone, 0.5% KH_2_PO_4_, 0.05% MgSO_4_ 7×H_2_O, and 1.5% agar) at 28°C. Oligonucleotides and plasmids are listed in Table S4.

### CRISPR/Cas9 plasmid constructions for gene deletions.

For the design of the deletion constructs, two protospacer sequences per gene were chosen to produce two different sgRNAs concurrently from the respective constructs. The strategy is based on our previous work. To insert two different protospacer sequences into linearized pFC332 vector, two chimeric PCR fragments obtained through fusing two small PCR fragments, respectively, were combined in a NEBuilder reaction (New England Biolabs, Frankfurt, Germany). In detail, two small PCR fragments (f1 and f2), to insert the first protospacer sequence, were amplified using pFC334 as the template; another two small PCR fragments (f3 and f4), to insert the second protospacer sequence, were amplified using pAK4 as the template. Next, two chimeric fragments F1 and F2 were obtained by combining f1/f2 and f3/f4 in a fusion-cloning step and then used to insert two different protospacer sequences into the pFC332 vector. The primers, which contain the variable regions, used to generate the sgRNA gene fragments were obtained from MWG Eurofins and are listed in Table S4. The fragments were amplified from pFC334 and pAK4 with proofreading polymerase Q5 (NEB) by a touchdown PCR program (denaturation, initial step for 3 min at 98°C; all following denaturation steps for 20 s at 98°C; annealing, 5 cycles at 67°C for 20 s, 5 cycles at 65°C for 20 s, 25 cycles at 63°C for 20 s; and elongation, 10 s at 72°C). The chimeric fragments were amplified by a fusion-PCR program (denaturation, initial step for 3 min at 98°C; all following steps are 30 cycles; denaturation, 20 s at 98°C; annealing, 20 s at 63°C; and elongation, 25 s at 72°C). Standard reaction mixture volumes were 50 μL, including 1 U Q5 reaction buffer, 200 μM deoxynucleotide triphosphates (dNTPs), 0.5 μM primers, 1 U Q5, and 100 ng of plasmid DNA or PCR fragments. Plasmid pFC332 was linearized using PacI and assembled with the PCR fragments, following the NEBuilder protocol. Escherichia coli transformation and plasmid isolation were done according to standard protocols.

### Plasmid and chimeric cassette construction for PksA tagging.

A CRISPR/Cas9 knock-in strategy was used for PksA GFP tagging. For the design of the knock-in construct, one protospacer sequence near the stop codon of *pksA* was chosen. To insert the protospacer sequence into the linearized pFC332 vector, two PCR fragments, amplified with pFC334 as the template, were combined in a NEBuilder reaction (New England Biolabs, Frankfurt, Germany). The chimeric cassette *pksA*(L)-*GFP*-*pksA*(R) was obtained through a fusion-PCR program. In detail, two 600-bp fragments of the upstream or downstream region of the Cas9-cutting site in the *pksA* gene, *pksA*(L) and *pksA*(R), were amplified using A. alternata genomic DNA as the template. GFP was amplified using pBV1 as the template. Finally, the chimeric cassette *pksA*(L)-*gfp*-*pksA*(R) was obtained in a fusion-cloning step. The oligonucleotides are listed in Table S4.

### Plasmid construction for peroxisomal labeling.

The *gpdA*(*p*) and *trpC*(*t*) fragments were amplified from plasmid pBV1. The *mCherry-SKL* fragment was amplified from plasmid pVW05. The chimeric fragment was obtained by combination of *gpdA*(*p*), *mCherry-SKL*, and *trpC*(*t*) fragments in a fusion-cloning step and then cloned into the linearized vector pJet-*hph* (hygromycin B phosphotransferase gene) in a NEBuilder reaction. The primers are listed in Table S4.

### Heterologous expression of A. alternata *pksA*.

The open reading frame (ORF) of the *pksA* gene was amplified by PCR using the primers pksA-AO-fw/rev (Table S4) and A. alternata genomic DNA as the template. The PCR fragment was cloned into the linearized vector pTYGSade2.0 (Table S4) in a NEBuilder reaction.

### Laccase-tagging plasmid constructions.

The construction of laccase-tagging plasmids was based on the strategy tagging laccase Abr2 in A. fumigatus (58). The *lcc*(*p*)-*lcc* (laccase-encoding gene promoter and ORF sequences) fragment was amplified using A. alternata genomic DNA as the template. GA5-*mCherry* fragment was amplified from the plasmid pVW05. The *trpC*(*t*) fragment was amplified from the plasmid pBV1. The chimeric fragment *lcc*(*p*)-*lcc*-GA5-*mCherry*-*trpC*(*t*) was obtained by the combination of *lcc*(*p*)-*lcc*, GA5-*mCherry*, and *trpC*(*t*) fragments in a fusion-cloning step and then cloned into the linearized vector pJet-*hph* (hygromycin B phosphotransferase gene) in a NEBuilder reaction. The primers used to generate *lcc*(*p*)-*lcc*-GA5-*mCherry*-*trpC*(*t*) fragment are listed in Table S4.

### Complementation of A. alternata *pksA*.

The *pksA* gene sequences containing 1 kb upstream of the ORF (promoter), ORF, and 1 kb downstream of the ORF (terminator) were amplified by PCR using A. alternata genomic DNA as the template. The PCR fragment was cloned into the linearized vector pJet-*hph* in a NEBuilder reaction. The primers are listed in Table S4.

### Promoter-reporter plasmids.

The *pksA*(*p*), *aygA*(*p*), and *aygB*(*p*) (promoter sequences of the PksA-, AygA-, and AygB-encoding genes) fragments were amplified using A. alternata genomic DNA as the template. *mCherry*-*stuA*-*trpc*(*t*) and *gfp*-*stuA*-*trpc*(*t*) fragments were amplified using pVW05 and pBV1 as the templates, respectively. *pksA*(*p*)*-mCherry*-*stuA*-*trpc*(*t*), *aygA*(*p*)*-gfp*-*stuA*-*trpc*(*t*), and *aygB*(*p*)*-gfp*-*stuA*-*trpc*(*t*) were obtained by the combination of *pksA*(*p*)/*aygA*(*p*)/*aygB*(*p*) and *mCherry*-*stuA*-*trpc*(*t*)/*gfp*-*stuA*-*trpc*(*t*) fragments in a fusion-cloning step and then cloned into the linearized vector pJet-*hph* in a NEBuilder reaction. The primers are listed in Table S4.

### Protoplast transformation of A. alternata.

Fresh spores were harvested from a mCDB culture plate and inoculated into 100 mL of mCDB medium for overnight cultivation at 28°C and 180 rpm. The mycelia were harvested by filtering, washed once with 0.7 M NaCl, and then digested in a Kitalase (Wako Chemicals) suspension (150 mg in 15 mL of 0.7 M NaCl) for 1 h with soft shaking at 100 rpm and 30°C. The quality and quantity of protoplast were checked using microscopy. Protoplasts were separated from cell fragments by filtering through two layers of Miracloth (Millipore) and precipitated at 2,430 rpm for 10 min at room temperature. The Kitalase solution was discarded, and the protoplasts were washed once with ice-cold 0.7 M NaCl through repeating the centrifugation step at 4°C. Then, the protoplasts were resuspended in 200 μL of STC solution (1 M sorbitol, 50 mM CaCl_2_, 50 mM Tris-HCl, pH 8.0), and 5 μg of plasmid DNA were added to the protoplasts followed by an incubation on ice for 10 min. DNA uptake was induced with a heat shock at 42°C for 5 min, and after a 10-min incubation step on ice, 1 mL of 40% polyethylene glycol 4000 (PEG 4000) solution (50 mM Tris-HCl, pH 8.0, and 50 mM CaCl_2_) was added to the protoplasts, followed by 20 min of incubation at room temperature. The suspension was mixed with 50 mL of warm regeneration medium (34.3% saccharose, 0.5% yeast extract, 0.5% casein hydrolysate, 0.75% agar) and split into a petri dish with 15 cm diameter. After overnight incubation at 28°C, the transformation plate was overlaid with 40 mL of warm regeneration medium containing HygB (80 μg/mL) and again incubated at 28°C until colonies were formed.

### Protoplast transformation of A. oryzae.

A. oryzae strain NSAR1 was grown on mCD medium for 7 days at 28°C. Fresh spores were harvested and inoculated overnight in liquid mCD medium at 28°C and 180 rpm. The mycelium was filtered through Miracloth and washed with 0.8 M NaCl solution. The mycelium was digested in a Trichoderma harzianum lysing enzyme (Novozymes) for 1 h with soft shaking at 30°C and 100 rpm. The quality and quantity of protoplast were checked using microscopy. The protoplasts were separated by filtering through two layers of Miracloth and precipitated for 5 min at 4°C. The lysing enzyme solution was discarded, and protoplasts were resuspended in 100 μL of solution 1 (0.8 M NaCl, 10 mM CaCl_2_, 50 mM Tris-HCl, pH 7.5). A total of 3 μg of plasmid DNA were add to the protoplasts and incubated on ice for 5 min. Then, 1 mL solution 2 (40% PEG 4000, 0.8 M NaCl, 50 mM Tris-HCl, pH 7.5, and 50 mM CaCl_2_) was added to the protoplasts, followed by 20 min of incubation at room temperature. The suspension was mixed with 15 mL of warm CZDS soft-top agar (3.5% Czapek-Dox broth, 0.7% agar, and 1 M sorbitol) and poured onto Czapek-Dox (3% sucrose, 0.3% NaNO_3_, 0.1% KH_2_PO_4_, 0.05% KCl, 0.05% MgSO_4_ 7×H_2_O, 0.001% FeSO_4_ 7×H_2_O, 1.5% agar, pH 7.3) plate supplemented with 1 M sorbitol, arginine, and methionine and incubated at 28°C until colonies were formed.

### Microscopy.

For microscopic analysis, A. alternata strains were grown on mCDB medium for 3 to 5 days at 28°C. For the promoter-reporter assay, a slice of agar containing spores, aerial hypha, and substrate hypha was obtained by trimming the A. alternata colony using a razor blade. Then, it was placed on a microscope slide and covered with a coverslip. Conventional fluorescence images were captured using an EC Plan-Neofluar 100×/1.3 oil or 40×/0.75 objective with an AxioImager Z.1 and AxioCamMR (Zeiss).

### Melanin content measurement.

Fresh spores of wild-type strain were inoculated in 20 mL of liquid mCDB medium or mCDB medium containing tomato puree (400 g/liter) in a Ø 15-cm petri dish for 7 days at 28°C. Mycelia of respective strains were collected and frozen using liquid nitrogen. The frozen mycelia were ground into powder, suspended in 5 mL of 1 M NaOH solution, and then boiled at 100°C for 2 h. The pH value of the solution was adjusted to 2.0 with 5 M HCl, and the acidified solution was centrifuged at 10,000 × *g* for 20 min. The resulting melanin (supernatant) was dissolved in 2% NaOH, and then the solution was measured using a spectrophotometer for absorbance at 459 nm (70).

### RNA isolation and quantitative real-time PCR.

A. alternata spores (10^5^) were inoculated in 10 mL of mCDB or mCDB containing tomato puree (400 g/liter) in a petri dish of 3.5 cm diameter and incubated for 5 days at 28°C. Subsequently, the mycelia were harvested and frozen immediately in liquid nitrogen. Frozen mycelia were ground into powder, and total RNA was isolated using a fungal RNA extraction kit (Omega). RNA samples were quantified and treated with TURBO DNA-free kit (Invitrogen) to remove DNA contaminations. After the treatment, the isolated RNA was diluted to 50 ng/μL. SensiFAST SYBR and fluorescein one-step kit from Bioline (Luckenwalde) was used for quantitative real-time PCR. Each reaction mixture of 20 μL contained 1 μL of reverse transcriptase (RT) enzyme, 0.16 μM primers, and 100 ng of totally RNA. The program included 10 min of the reverse transcription reaction at 50°C, followed by 5 min at 95°C to inactive the reverse transcriptase and 40 PCR cycles (10 s at 95°C and then 30 s at 59°C). After each PCR, melting curve analyses were performed to show the specific amplification of single DNA segments and the absence of nonspecifically amplified DNA. The histone gene *H2B* was used to normalize the results of each gene. Each expression level is the average of three biological replicates. The error bar indicates the standard deviation for the replicates. The oligonucleotides used in this study are listed in Table S4.

### Metabolite extraction and thin-layer chromatographic (TLC) analysis.

To extract secondary metabolites, A. alternata and A. oryzae strains were grown for 1 to 7 days on their respective production media at 28°C. Four disks from each plate were excised with the back of a blue pipette tip and extracted by shaking with 1 mL of ethyl acetate for 1 h. After centrifugation at 13,000 rpm for 10 min, 700 μL of supernatant containing secondary metabolites of each sample was token into a 1.5-mL microtube and dried at room temperature, followed by dissolving secondary metabolites again using 70 μL of ethyl acetate for further TLC analysis or dissolving in 1 mL ACN:H_2_O (1:1, vol/vol) for further LC-HRMS analysis.

For the qualitative analysis of the A. alternata extracts, 10 μL of crude extract was put on TLC plates coated with 0.25-mm silica gel as stationary phase (precoated TLC plates SIL G-25, Macherey-Nagel, Düren, Germany). The mobile phase for separation of the metabolites consisted of 50% of toluol, 40% of ethyl acetate, and 10% of formic acid. The secondary metabolites were visualized using UV light at 254 nm.

### LC-HRMS analysis of A. alternata and A. oryzae samples.

For analysis of A. alternata secondary metabolites, the respective bands were extracted and analyzed with LC-HRMS. The bands were visualized with UV light (254 nm), scratched off the TLC plates, and collected in reaction tubes. To obtain more intense signals, five bands were pooled for each metabolite; 500 μL of a 1:1 (vol/vol) mixture of MS grade acetonitrile (ACN, HiPerSolv Chromanorm, HPLC gradient grade, VWR, Vienna, Austria) and water (ELGA Purelab Ultra-AN-MK2 system, Veolia Water, Vienna, Austria) were added, mixed, and kept at room temperature for 30 min. After filtration (0.2-μm polytetrafluoroethylene [PTFE], 13-mm syringe filters, VWR International), the extracts were transferred to glass vials for measurement.

To search for initially released PksA polyketide products, dried A. oryzae extracts were dissolved in 1 mL ACN:H_2_O (1:1, vol/vol) and further diluted (1:10,000, vol/vol) for Fullscan LC-HRMS measurement. For MS/MS spectra generation, 1:1,000 (vol/vol) dilutions were prepared.

LC-HRMS measurements were carried out with a Vanquish UHPLC system coupled to a QExactive HF Orbitrap via heated ESI source operating in polarity switching mode (all Thermo Fisher Scientific, Bremen, Germany) applying the chromatographic method described previously (71). Full scan mass spectra were recorded with a resolving power setting of 120,000 (FWHM, full width at half maximum) at *m*/*z* 200 and with mass ranges between *m*/*z* 100 and 1,000. MS/MS fragmentation was carried out using an inclusion list of the defined targets, and collision energy was adapted for the respective target (either stepped CE [20, 45, 70] or CE 70, 80, or 90). For identification reference standards of ATX I, ATX II, and ALP were used. In addition, T4HN was obtained from MedChemExpress.

The data were evaluated with the vendor software Xcalibur. The metabolites stated as identified were compared and confirmed with the authentic reference standards and classified as level 1 according to the metabolomics standards initiative. Other described compounds are annotated with level 3 (72).
