# Supplementary figures and images for: Fungal Melanin Biosynthesis Pathway as Source for Fungal Toxins
Source: mBio. 2022 Apr 27;13(3):e00219-22. doi: 10.1128/mbio.00219-22 (PMC9239091; doi:10.1128/mbio.00219-22)

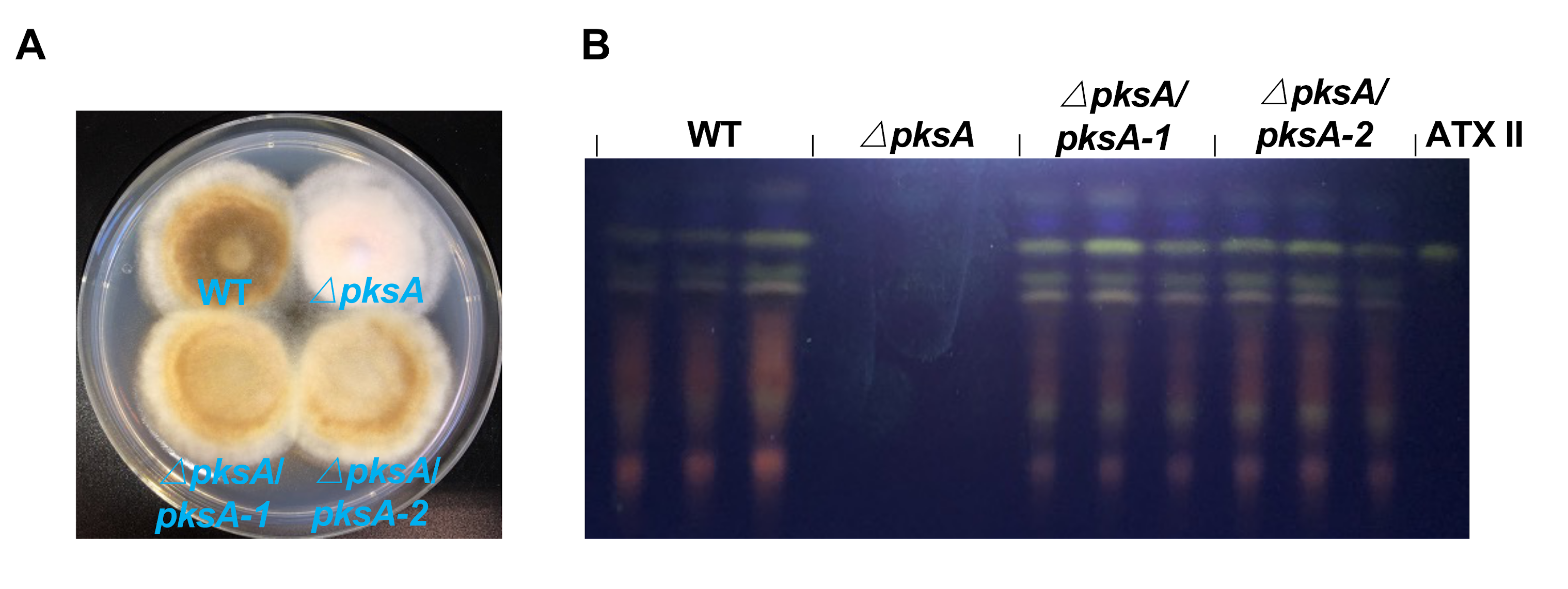

Supplement: FIG S1 [file mbio.00219-22-s0001.tif]

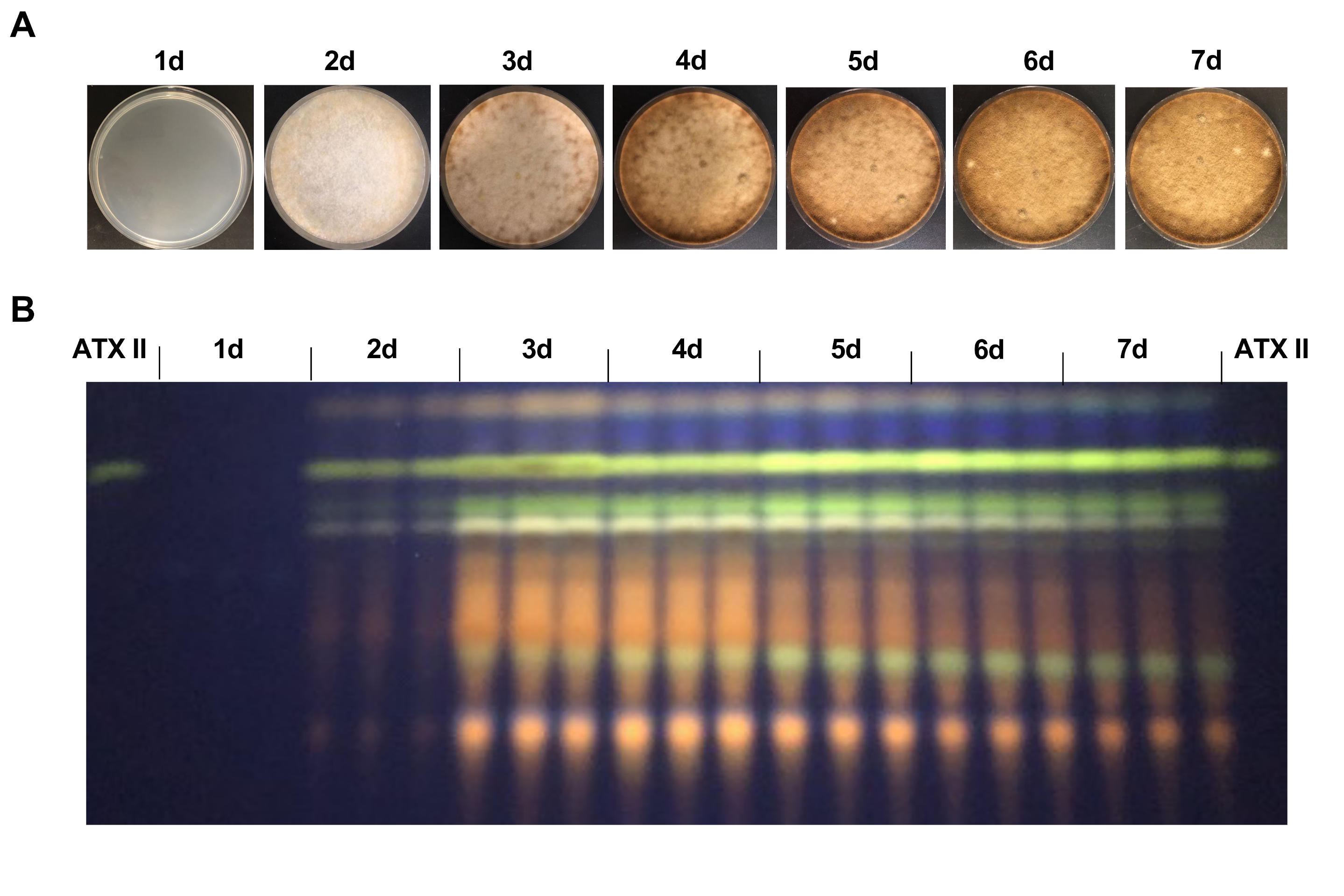

Supplement: FIG S2 [file mbio.00219-22-s0002.tif]

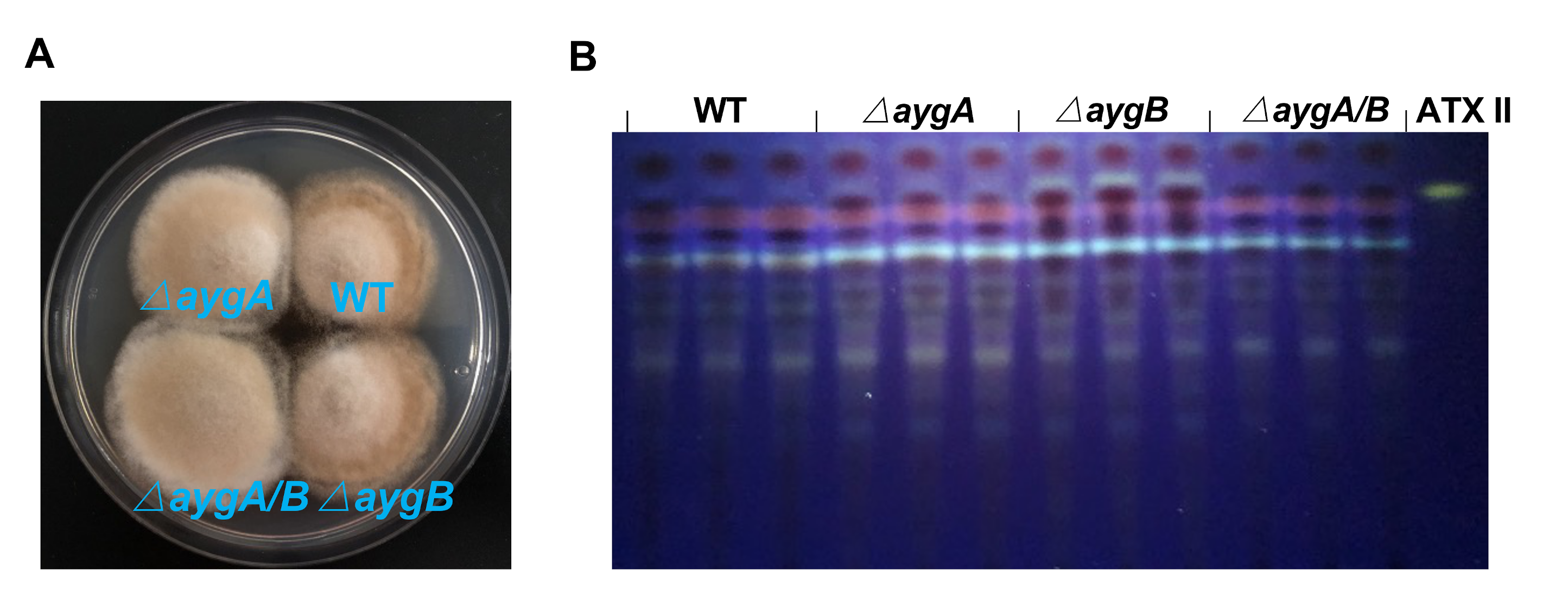

Supplement: FIG S3 [file mbio.00219-22-s0003.tif]

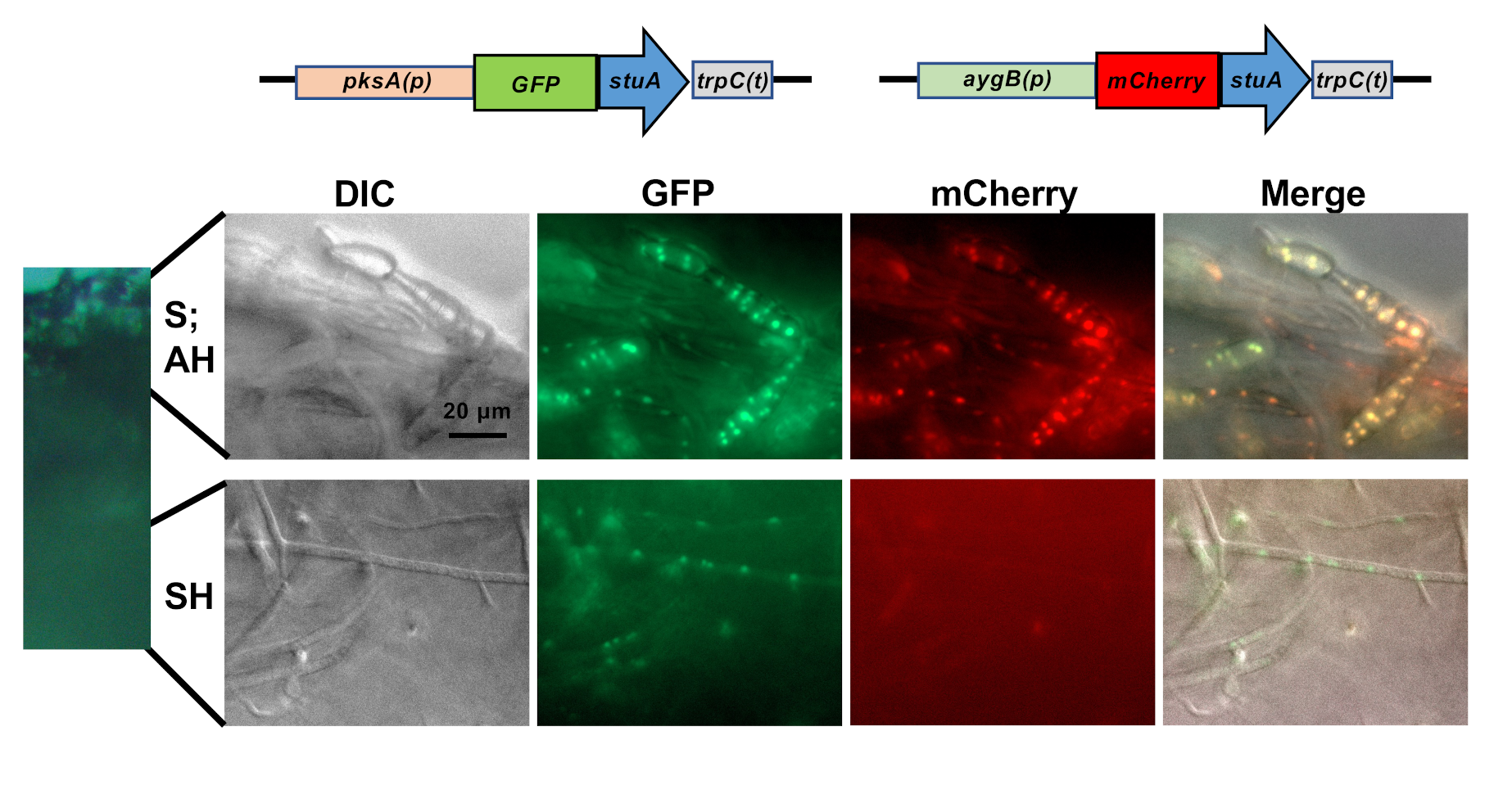

Supplement: FIG S4 [file mbio.00219-22-s0004.tif]

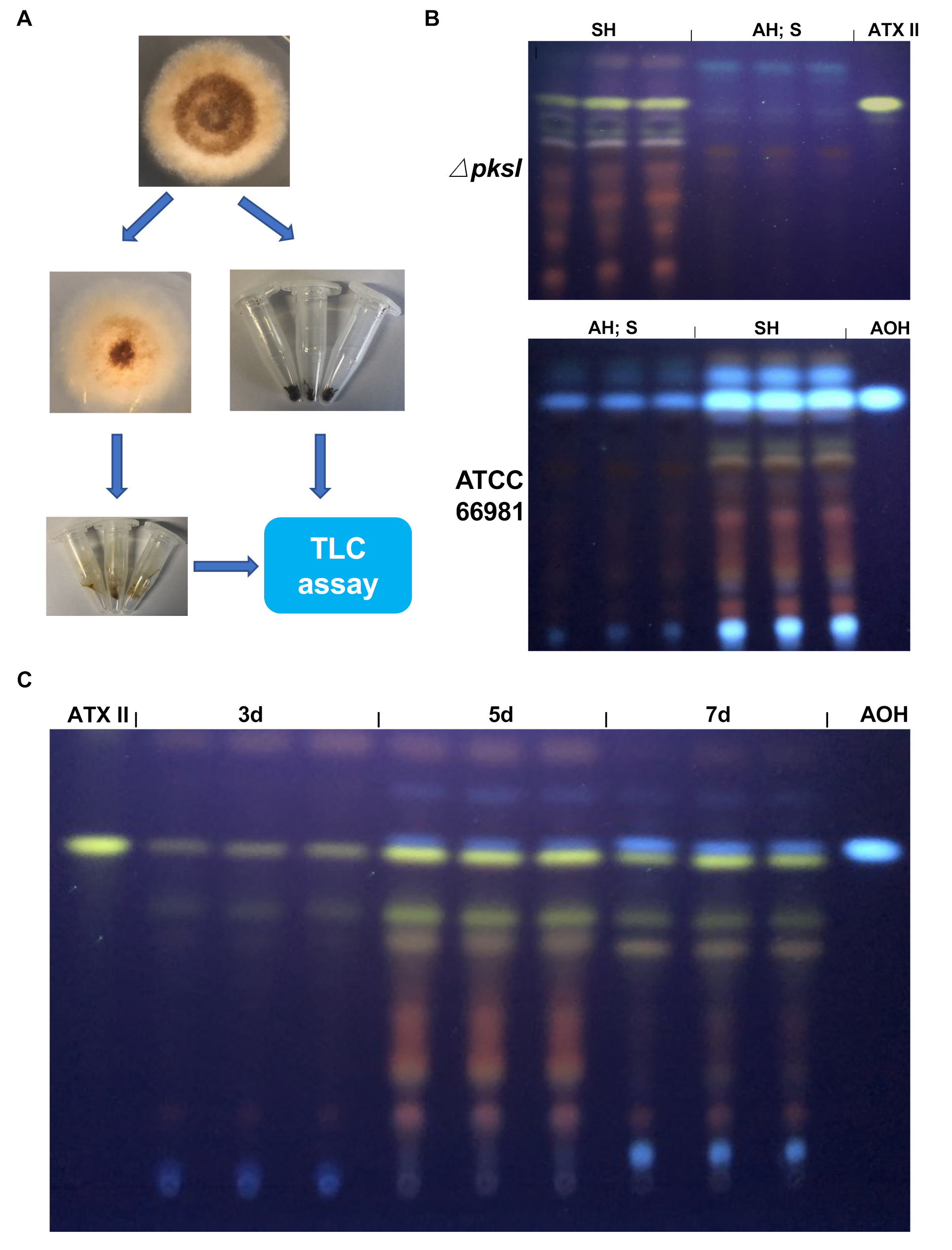

Supplement: FIG S5 [file mbio.00219-22-s0005.tif]

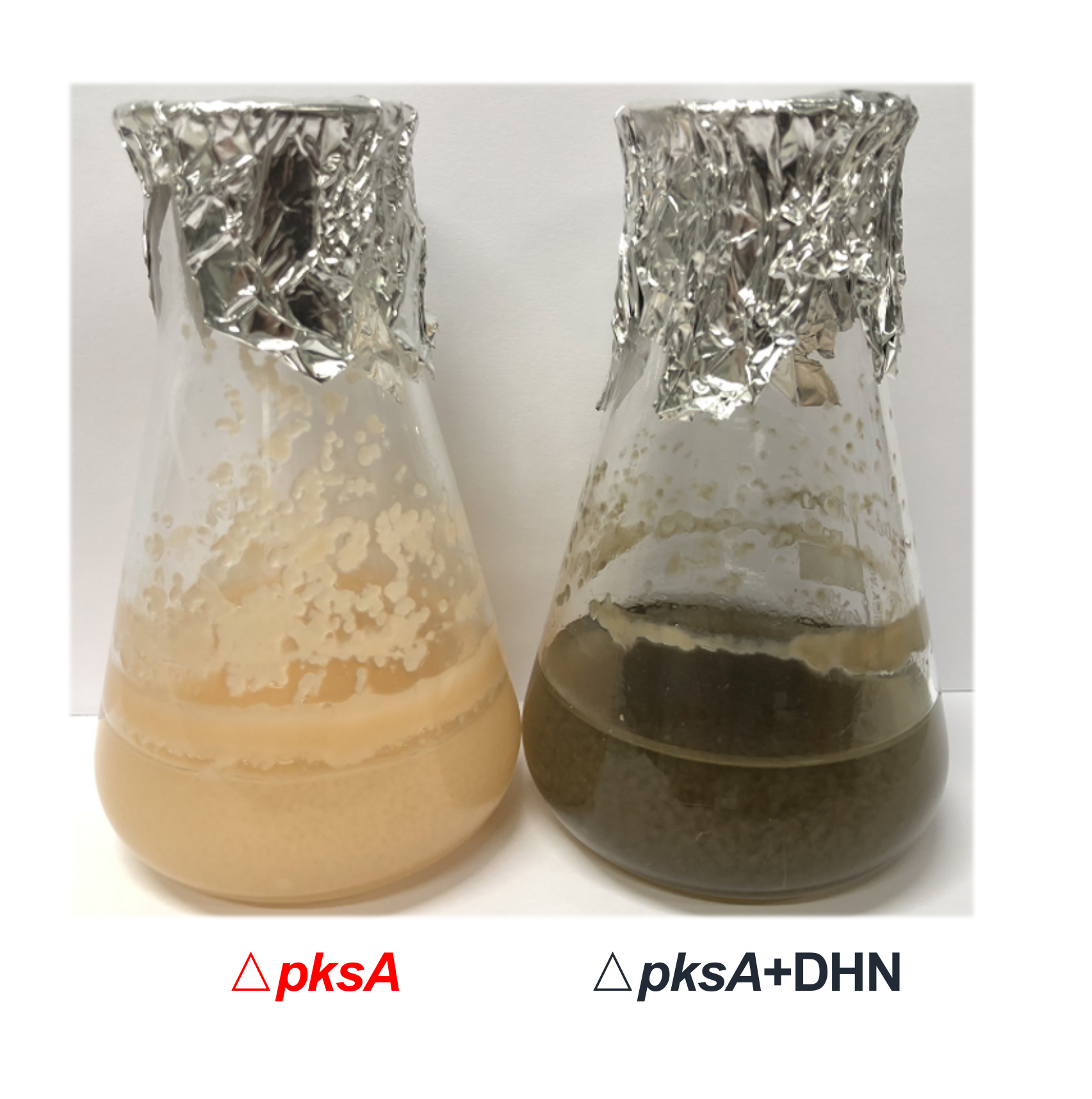

Supplement: FIG S6 [file mbio.00219-22-s0006.tif]
