# Supplementary material for: Fungal Melanin Biosynthesis Pathway as Source for Fungal Toxins
Source: mBio. 2022 Apr 27;13(3):e00219-22. doi: 10.1128/mbio.00219-22 (PMC9239091; doi:10.1128/mbio.00219-22)
Supplement: TABLE S1 [file mbio.00219-22-s0007.pdf]

Table S1: Detailed information for the identification of ATX II, ALP and ATX I. Retention time, detection ions, accurate mass of the most abundant ion as well as mass deviation are shown for the reference standards and the respective biological sample from TLC plates.

**Name**	**Molecular Formula**		**Retention time [min]**	**Detected Ions**	**Accurate Mass of Most Abundant Ion [*m/z*]**	**Mass Deviation [ppm]**
ATX II	$C_{20}H_{14}O_6$	reference standard	23.09	[M-H]- *, [M+Cl]-	349.0719	0.40
		sample	23.10	[M-H]- *	349.0718	0.11
ALP	$C_{20}H_{14}O_6$	reference standard	20.97	[M-H]- *; [M+Cl]-; [M+FA-H]-	349.0719	0.40
		sample	20.96	[M+H]+; [M-H]- *; [M+Cl]-; [M+FA-H]-	349.0717	-0.17
ATX I	$C_{20}H_{16}O_6$	reference standard	20.69	[M-H]- *; [M+Cl]-; [M+FA-H]-	351.0877	0.82
		sample	20.67	[M+H]+; [M-H]- *; [M+Cl]-; [M+FA-H]-	351.0874	-0.03

* most abundant ion
