# Supplementary material for: Fungal Melanin Biosynthesis Pathway as Source for Fungal Toxins
Source: mBio. 2022 Apr 27;13(3):e00219-22. doi: 10.1128/mbio.00219-22 (PMC9239091; doi:10.1128/mbio.00219-22)
Supplement: TABLE S2 [file mbio.00219-22-s0008.pdf]

Table S2: Gene annotation according to https://mycocosm.jgi.doe.gov/Alalte1/Alalte1.home.html. The introns were confirmed by RNAseq.

Gene (Transcript ID)	Gene size (bp)	Introns (location)	Amnio acids (aa)
pksA (111952)	6539	53 (6442-6494)	2162
aygA (115293)	1343	80 (106-185)	421
aygB (105009)	1212	0	404
brm1 (105968)	654	47 (38-84), 49 (141-189)	186
brm2 (111954)	904	51 (62-112), 49 (590-638)	268
brm3 (112254)	807	0	269
cmrA (111953)	3273	100 (71-170), 84 (226-309), 56 (2161-2216)	1011
lccA (*112523*)	1963	226 (1504-1729)	579
lccB(114657)	2104	49 (358-406), 53 (608-660), 138 (1061-1198), 52 (1633-1684)	604
lccC (115320)	1847	50 (229-278)	599
lccD (114031)	1864	61 (261-321), 63 (468-530), 54 (737-790)	562
lccE (111569)	2321	49 (765-813), 49 (1011-1059)	741
lccF (110245)	1822	89 (97-185), 47(572-618)	562
lccG (116332)	2070	0	690
