# Supplementary material for: Fungal Melanin Biosynthesis Pathway as Source for Fungal Toxins
Source: mBio. 2022 Apr 27;13(3):e00219-22. doi: 10.1128/mbio.00219-22 (PMC9239091; doi:10.1128/mbio.00219-22)
Supplement: TABLE S3 [file mbio.00219-22-s0009.pdf]

Table S3: ***Alternaria alternata*** **and** ***Aspergillus oryzae*** **strains used in this study.**

Strain	Genotype or description	Source
Alternaria alternata		
ATCC 66981	Parental strain	Christopher Lawrence, Virginia Tech, Blacksburg, VA, USA
⊿pksA	*pksA* deleted in parental strain	(1)
SMW35	*⊿pksI*, considered as wild type	(2)
SJG9	*⊿lccD*	This study
SJG10	*⊿lccC*	This study
SJG11	*⊿lccB*	This study
SJG14	*⊿lccF*	This study
SJG21	*⊿brm3*	This study
SJG22	*⊿brm2/3*	This study
SJG25	*⊿aygB*	This study
SJG26	*⊿aygA*	This study
SJG27	*⊿aygA/B*	This study
SJG28	*⊿pksA*	This study
SJG29	*⊿cmrA*	This study
SJG37	*pksA(p)::GFP::stuA::trpC(t),* checking the expression of *pksA* in wild type strain	This study
SJG38	*pksA(p)::GFP::stuA::trpC(t)* checking the expression of *pksA* in *⊿cmrA* strain	This study
SJG39	*pksA(p)::GFP::stuA::trpC(t)* *aygA(p)::mCherry::stuA::trpC(t)* checking the expression of *pksA* and *aygA* in wild type strain	This study
SJG40	*pksA(p)::GFP::stuA::trpC(t)* *aygB(p)::mCherry::stuA::trpC(t)* checking the expression of *pksA* and *aygB* in wild type	This study

	strain	
SJG42	*lccB(p)::lccB::mCherry::trpC(t)*	This study
SJG43	*lccC(p)::lccC::mCherry::trpC(t)*	This study
SJG44	*lccD(p)::lccD::mCherry::trpC(t)*	This study
SJG45	*lccF(p)::lccF::mCherry::trpC(t)*	This study
SJG57	*⊿brm1*	This study
SJG58	*⊿brm2*	This study
SJG59	*⊿lccC/D*	This study
SJG60	*⊿lccB/F*	This study
SJG62	*⊿pksA* complemented with *A. alternata pksA*	This study
SJG63	*pksA(p)::pksA::gfp::pksA(t) gpdA(p)::mCherry-SKL::trpC(t)*	This study
Aspergillus oryzae		
NSAR1	*niaD⁻, sC⁻, ⊿argB, adeA⁻*	Russel J. Cox, Leibniz Universität Hannover
SJG61	*amyB(p)::pksA::amyB(t); adh1(p)::adh1(t); gpdA(p)::gpdA(t); enoA(p)::enoA(t); ampR; adeA; URA3;* expressing *A. alternata pksA* in *A. oryzae*	This study

1. Wenderoth M, Pinecker C, Voß B, Fischer R. 2017. Establishment of CRISPR/Cas9 in *Alternaria alternata*. Fungal Genet Biol 101:55–60.
2. Wenderoth M, Garganese F, Schmidt-Heydt M, Soukup ST, Ippolito A, Sanzani SM, Fischer R. 2019. Alternariol as virulence and colonization factor of *Alternaria alternata* during plant infection. Mol Microbiol 112:131–146.

Proof of mutant strains.

Mutants	PCR Testing Results	Sequencing Results	Deletion Region
△*pksA*	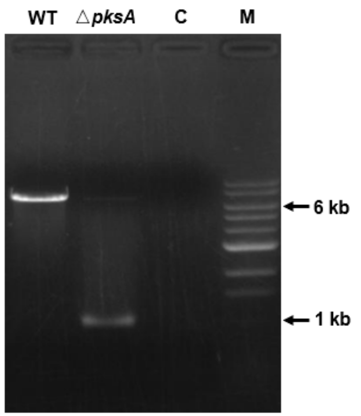	***pksA*** ATGAACGTCCTCATTTTCGGAGATCAAACCGCCGACCAGTACCCGCTCCTGCGG<br>**△*pksA*** ATGAACGTCCTCATTTTCGGAGATCAAACCGCCGACCAGTACCCGCTC------------<br>***pksA*** *****AACTGGTCAACTCTGGTGAGAAGGTTGAGAACCTC*************AAGCTATAA<br>**△*pksA*** --AGAAGGTTGAGAACCTC*************AAGCTATAA	5965-bp deletion: 5965 bp of the ORF
△*aygB*	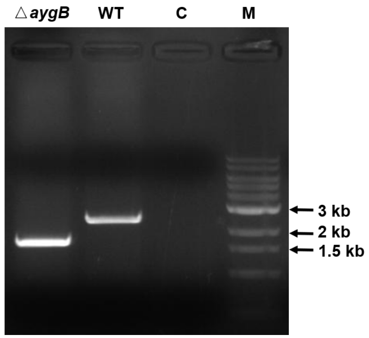	***aygB*** ATGCCTAACGGAAACTGGATTCTTGGCGACCTCTTCACAAAGGACTACGGACAT<br>**△*aygB*** ATGCCTAACGGAAACTGGATTCTTGGCGACCTCTTCA-----------------------------------<br>***aygB*** *****ACGGTGTATCCGTGGATGGAAGAGGTTATGGCGAGTAAGTAGATATCTGGT<br>**△*aygB*** ---AGATATCTGGT	1173-bp deletion: 1173 bp of the ORF

△aygA

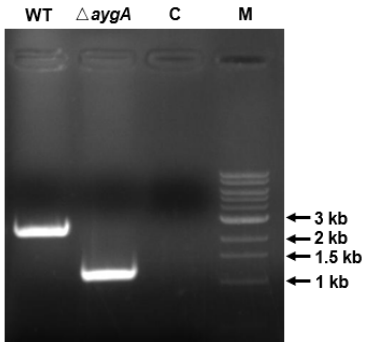

aygA ATGGCAG******ATGAGTCTTACGAACAGCTTTGGGAGACGAAGTGGAAGAAGCCT
△aygA ATGGCAG******ATGAGTCTTACGAAC--
aygA *****GGCTGTATAAGCTGTTTGGTATTGAGGCCGAAGTCGGGGC******AAGTACTAA
△aygA --GGGGC******AAGTACTAA

1218-bp deletion: 1218 bp of the ORF

△aygA (△aygB/ aygA double mutant)

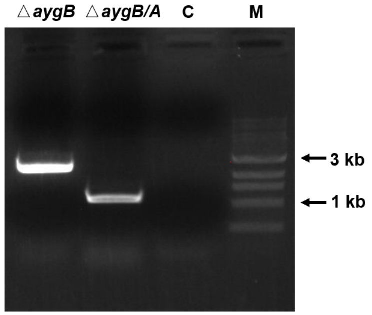

aygA ATGG******ACTATGAGTCTTACGAACAGCTTTGGGAGACGAAGTGGAAGAAGCCT
△aygA ATGG******ACTA--
aygA *****GGCTGTATAAGCTGTTTGGTATTGAGGCCGAAGTCGGGGC******AAGTACTAA
△aygA --TGAGGCCGAAGTCGGGGC******AAGTACTAA

1226-bp deletion: 1226 bp of the ORF

△brm1

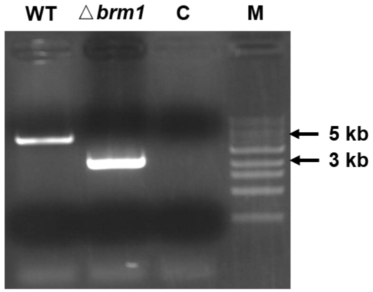

brm1 GGTGTAGAAATATTTCGG**
△brm1 GGTGTAGAAATATT--
brm1 *****TCATGTTTGAGAAGAACGAATTGAAGCCTACGTTCGAGGGTTA************
△brm1 --
brm1 *****ACAGCACTTCATCGGCGGTACACGGTGGG****************CAGGCGTTGTAG
△brm1 --
brm1 ********************************GATCTATCGACCATGTCATTCAACAACAGCTACGGC
△brm1 ---TATCGACCATGTCATTCAACAACAGCTACGGC

2542-bp deletion: 279 bp of the 5' UTR, 654 bp of the ORF and 1609 bp of the 3' UTR

△*brm2*	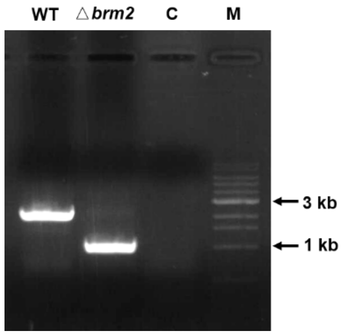	***brm2*** GTTTGCAGGCTAAACCTT**<br>**△*brm2*** GTTTGCAGGCTAAAC--<br>***brm2*** *****ATGGCATCAATCGAGCAGACATGGAGCCTTGCCGGCAAGGTC************<br>**△*brm2*** ---<br>***brm2*** *****CTACAATACTAACTATCACCCAGGCCGT******************GCTTGCATGTAA<br>**△*brm2*** -----------------------TAACTATCACCCAGGCCGT******************GCTTGCATGTAA	925-bp deletion: 98 bp of the ORF and 827 bp of the 5' UTR
△*brm3*	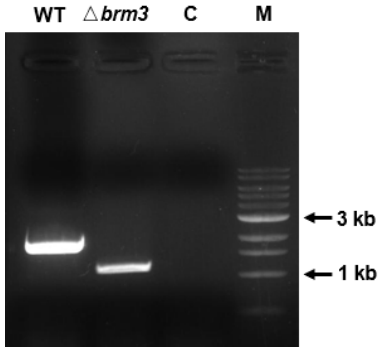	***brm3*** AGCTTCGGGCGCGAAGGTGTAGAGCCGCAGGTCCCTGAAGCAGCTATTTTCGGT<br>**△*brm3*** AGCTTCGGGCGCGAAGGTGTAGAGCCGCAGGTCCCTGAAGCAGCTATTTTCGGT<br>***brm3*** GTTCGTTTCTTCGGATCCCCGCCCGCGGCCGCCTACCGTGAAGACCATCGTTTGA<br>**△*brm3*** GTTCGTTTCTTCGG---<br>***brm3*** *****CGGCCAAACCGCGTACAAGCACATTGAGGACAACG*********CGTCGCAGTAA<br>**△*brm3*** --ATTGAGGACAACG*********CGTCGCAGTAA	623-bp deletion: 411 bp of the ORF and 212 bp of the 5' UTR

△*brm3* (△*brm2/brm3 double mutant*)	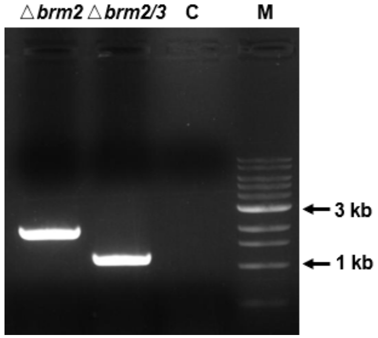 	***brm3*** AGCTTCGGGCGCGAAGGTGTAGAGCCGCAGGTCCCTGAAGCAGCTATTTTCGGT<br>△***brm3*** AGCTTCGGGCGCGAAGGTGTAGAGC--<br>***brm3*** *****CGGCCAAACCGCGTACAAGCACATTGAGGACAACG*********CGTCGCAGTAA<br>△***brm3*** --ATTGAGGACAACG*********CGTCGCAGTAA	666-bp deletion: 411 bp of the ORF and 255 bp of the 5' UTR
△*lccB*	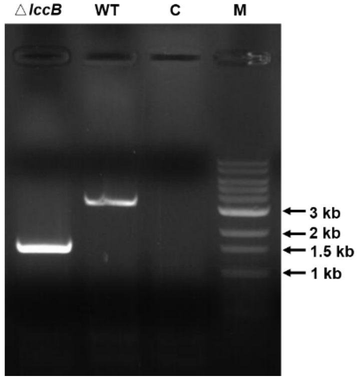 	***lccB*** ATGGTTG******CCCAGAGTTCCTGAGGTTCCGTGGAAAGAAACCGGTCTATGGAG<br>△***lccB*** ATGGTTG******CCCAGAGTTCCTGAGGTTCCGTGGAAAGA---------------------------------<br>***lccB*** *****TTTCTTGAGCGCTCGTCTGAGATTAAGGCCAAGGATGGAT******GGGCTATAG<br>△***lccB*** --------------------------------------TGAGATTAAGGCCAAGGATGGAT******GGGCTATAG	1864-bp deletion: 1864 bp of the ORF
△*lccC*	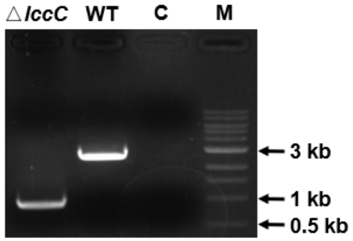 	***lccC*** ATGTTGG******ACTTTCCTTCGTATGAACAGATTGTCAAGAGGCAAGACGGTGCTT<br>△***lccC*** ATGTTGG******ACTTTCCTTCGTATGAACAGATTGTC---------------------------------------<br>***lccC*** *****TACTCAAGAATGGATGCGACGAGTGGGACTCTTACT*********CTGGTATCTAA<br>△***lccC*** --CGAGTGGGACTCTTACT*********CTGGTATCTAA	1657-bp deletion: 1657 bp of the ORF

△*lccD*

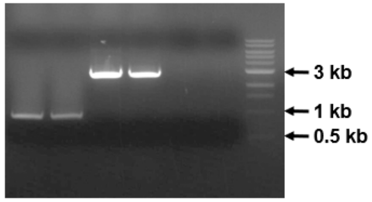

lccD	ATGCTCT******CACTTCGGATCTACGACCCAGAGCAGCCTGTGACGGAAACACTG
△*lccD*	ATGCTCT******CACTTCGGATCTACGACCCAGAGCAGC--
lccD	*****GACGAGTCATACCGAAACGGTCGGTATTGAGCAGGATGACTCCGGTGTTTAG
△*lccD*	--GGTCGGTATTGAGCAGGATGACTCCGGTGTTTAG

1741-bp deletion: 1741 bp of the ORF

△*lccF*

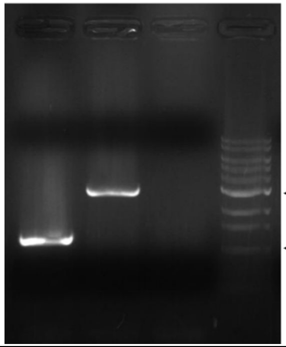

lccF	ATGTTGG******TGTAAGTACTCTTATTCCTAGTCACGACCAAAGGCGATAGGCCAC
△*lccF*	ATGTTGG******TGTAAGTACTCTTATTCCTAGTCA--
lccF	*****GCACTTTGCGATGCAGGAAAGAAGAAGTCGAGCGGTAAC******ATTGAGTAG
△*lccF*	--CGAGCGGTAAC******ATTGAGTAG

1596-bp deletion: 1596 bp of the ORF

△*cmrA*

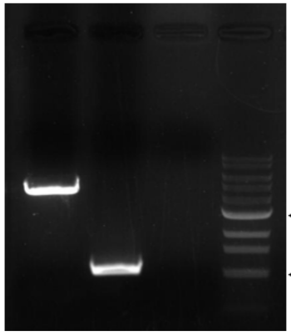

cmrA	ACATCTACAGCCAGGCTCTCTCCAGGACTACACGTCCAATCCGTGATTCAAAATGGTC
△*cmrA*	ACATCTACAGCCAGGCTC--
cmrA	*****GTGTCGCCACCAGGTTTTCCGATGGCATTCGTAGTAAACTC********GCGCGGTTAA
△*cmrA*	--GTAGTAAACTC********GCGCGGTTAA

3275-bp deletion: 3275 bp of the ORF

△*lccF* (△*lccB/lccF double mutant*)	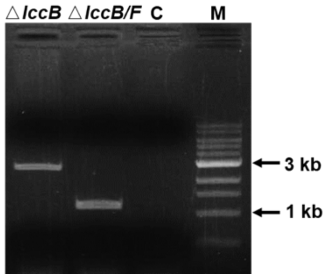	*lccF* ATGTTGG******TGTAAGTACTCTTATTCCTAGTCACGACCAAAGGCGATAGGCCAC<br>△*lccF* ATGTTGG******TGTAAGTACTCTTATTCCTAGTCACGA---<br>*lccF* *****GCACTTTGCGATGCAGGAAAGAAGAAGTCGAGCGGTAAC******ATTGAGTAG<br>△*lccF* ---CGAGCGGTAAC******ATTGAGTAG	1593-bp deletion: 1593 bp of the ORF
△*lccD* (△*lccC/lccD double mutant*)	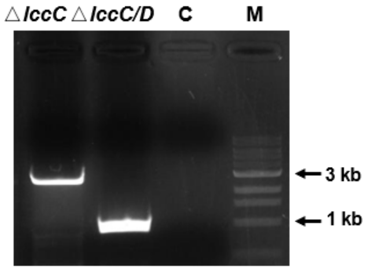	*lccD* ATGCTCT******CACTTCGGATCTACGACCCAGAGCAGCCTGTGACGGAAACACTG<br>△*lccD* ATGCTCT******CACTTCGGATCTACGACCCAGAGCAGCCTG------------------------------<br>*lccD* *****GACGAGTCATACCGAAACGGTCGGTATTGAGCAGGATGACTCCGGTGTTTAG<br>△*lccD* --GGTCGGTATTGAGCAGGATGACTCCGGTGTTTAG	1738-bp deletion: 1738 bp of the ORF

Annotation: The protospacers are underlined; the start codon (red); the stop codon (blue); 5' Un-translated Region (UTR) (gray); 3' UTR (gray); C: negative control; M: 1 kb gene ruler; WT: wild type.
