# Supplementary material for: Fungal Melanin Biosynthesis Pathway as Source for Fungal Toxins
Source: mBio. 2022 Apr 27;13(3):e00219-22. doi: 10.1128/mbio.00219-22 (PMC9239091; doi:10.1128/mbio.00219-22)
Supplement: TABLE S4 [file mbio.00219-22-s0010.pdf]

Plasmids

Name	Description	Reference
pFC332	*tef1*(p)*::cas9::tef1*(t); *gpdA*(p)*::gpdA*(t); *hph; ampR*; AMA1	Uffe H. Mortensen, Technical University of Denmark
pFC334	*tef1*(p)*::cas9::tef1*(t); *gpdA*(p)*::sgRNA-AnyA::gpdA*(t); *Afpyr4; ampR*; AMA1	Uffe H. Mortensen, Technical University of Denmark
pAK4	pJET1.2 blunt + *trpC*(p)*::sgRNA-AnyA::trpC*(t)	This work
pJET1.2 blunt	Cloning Vector, Component of CloneJET PCR Cloning Kit	Thermo Fisher Scientific, USA
pTYGSade2.0	*amyB*(p)::*amyB*(t); *adh1*(p)::*adh1*(t); *gpdA*(p)::*gpdA*(t); *enoA*(p)::*enoA*(t); ampR; adeA; URA3	Colin M. Lazarus, University of Bristol
pJG10	*tef1*(p)*::cas9::tef1*(t); *gpdA*(p)*::lccD-sgRNAcassette1::gpdA*(t)*::trpC*(p)*::lccD-sgRNAcassette2::trpC*(t); *hph*; *ampR*	This work
pJG11	*tef1*(p)*::cas9::tef1*(t); *gpdA*(p)*::lccC-sgRNAcassette1::gpdA*(t)*::trpC*(p)*::lccC-sgRNAcassette2::trpC*(t); *hph*; *ampR*	This work
pJG16	*tef1*(p)*::cas9::tef1*(t); *gpdA*(p)*::lccB-sgRNAcassette1::gpdA*(t)*::trpC*(p)*::lccB-sgRNAcassette2::trpC*(t); *hph*; *ampR*	This work
pJG19	*tef1*(p)*::cas9::tef1*(t); *gpdA*(p)*::lccF-sgRNAcassette1::gpdA*(t)*::trpC*(p)*::lccF-sgRNAcassette2::trpC*(t); *hph*; *ampR*	This work
pJG28	*tef1*(p)*::cas9::tef1*(t); *gpdA*(p)*::brm3-sgRNAcassette1::gpdA*(t)*::trpC*(p)*::brm3-sgRNAcassette2::trpC*(t); *hph*; *ampR*	This work
pJG30	*tef1*(p)*::cas9::tef1*(t); *gpdA*(p)*::aygB-sgRNAcassette1::gpdA*(t)*::trpC*(p)*::aygB-sgRNAcassette2::trpC*(t); *hph*; *ampR*	This work

pJG31	*tef1*(p)*::cas9::tef1*(t); *gpdA*(p)*::aygA-sgRNAcassette1::gpdA*(t)*::trpC*(p)*::aygA-sgRNAcassette2::trpC*(t); *hph*; *ampR*	This work
pAK1	*tef1*(p)*::cas9::tef1*(t); *gpdA*(p)*::pksA-sgRNAcassette1::gpdA*(t)*::trpC*(p)*::pksA-sgRNAcassette2::trpC*(t); *hph*; *ampR*	This work
pJG32	*tef1*(p)*::cas9::tef1*(t); *gpdA*(p)*::cmrA-sgRNAcassette1::gpdA*(t)*::trpC*(p)*::cmrA-sgRNAcassette2::trpC*(t); *hph*; *ampR*	This work
pMW89	*tef1*(p)*::cas9::tef1*(t); *gpdA*(p)*::brm1-sgRNAcassette1::gpdA*(t)*::trpC*(p)*::brm1-sgRNAcassette2::trpC*(t); *hph*; *ampR*	This work
pMW90	*tef1*(p)*::cas9::tef1*(t); *gpdA*(p)*::brm2-sgRNAcassette1::gpdA*(t)*::trpC*(p)*::brm2-sgRNAcassette2::trpC*(t); *hph*; *ampR*	This work
pJG33	pJET1.2 blunt + *hph*	This work
pJG37	*tef1*(p)*::cas9::tef1*(t); *gpdA*(p)*::pksA-sgRNAcassette3*; *hph*; *ampR*	This work
pJG38	*pksA*(p)*::gfp::stuA::*trpC(t); *hph*; *ampR*	This work
pJG40	*aygA*(p)*::mCherry::stuA::*trpC(t); *hph*; *ampR*	This work
pJG41	*aygB*(p)*::mCherry::stuA::*trpC(t); *hph*; *ampR*	This work
pJG43	*lccB*(p)::*lccB*::*mCherry*::*trpC*(t); *hph*; *ampR*	This work
pJG44	*lccC*(p)::*lccC*::*mCherry*::*trpC*(t); *hph*; *ampR*	This work
pJG45	*lccD*(p)::*lccD*::*mCherry*::*trpC*(t); *hph*; *ampR*	This work
pJG46	*lccF*(p)::*lccF*::*mCherry*::*trpC*(t); *hph*; *ampR*	This work
pJG47	*amyB*(p)::*pksA::amyB*(t); *adh1*(p)::*adh1*(t); *gpdA*(p)::*gpdA*(t); *enoA*(p)::*enoA*(t); ampR; adeA; URA3	This work
pJG48	*pksA*(p)*::pksA::pksA*(t); hph; *ampR*	This work
pJG59	*gpdA*(*p*)*::mCherry-SKL::trpC*(*t*) ; hph; *ampR*	This work
